# Recent Advances in Rapid Synthesis of Non-proteinogenic Amino Acids from Proteinogenic Amino Acids Derivatives via Direct Photo-Mediated C–H Functionalization

**DOI:** 10.3390/molecules25225270

**Published:** 2020-11-12

**Authors:** Zhenbo Yuan, Xuanzhong Liu, Changmei Liu, Yan Zhang, Yijian Rao

**Affiliations:** 1 Key Laboratory of Carbohydrate Chemistry and Biotechnology, Ministry of Education, School of Biotechnology, Jiangnan University, Wuxi 214122, China; zhenboyuan1992@jiangnan.edu.cn (Z.Y.); 6190201080@stu.jiangnan.edu.cn (X.L.); cmliu@jiangnan.edu.cn (C.L.); 2 School of Pharmaceutical Science, Jiangnan University, Wuxi 214122, China; zhangyan@jiangnan.edu.cn

**Keywords:** non-proteinogenic amino acids, photo-mediated, C–H functionalization

## Abstract

Non-proteinogenic amino acids have attracted tremendous interest for their essential applications in the realm of biology and chemistry. Recently, rising C–H functionalization has been considered an alternative powerful method for the direct synthesis of non-proteinogenic amino acids. Meanwhile, photochemistry has become popular for its predominant advantages of mild conditions and conservation of energy. Therefore, C–H functionalization and photochemistry have been merged to synthesize diverse non-proteinogenic amino acids in a mild and environmentally friendly way. In this review, the recent developments in the photo-mediated C–H functionalization of proteinogenic amino acids derivatives for the rapid synthesis of versatile non-proteinogenic amino acids are presented. Moreover, postulated mechanisms are also described wherever needed.

## 1. Introduction

**Figure 1:**
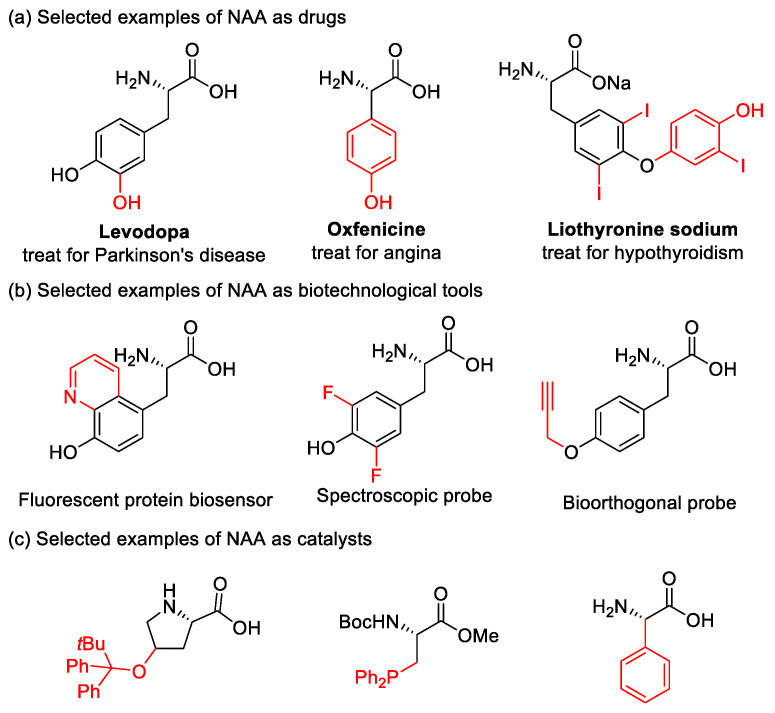
Selected examples of non-proteinogenic amino acids (NPAAs) as drugs (**a**), biotechnological tools (**b**) and catalysts (**c**).

Amino acids (AAs) are an important class of compounds containing amino groups and carboxyl groups at the same time and also fundamental building blocks of proteins responsible for life activities [1,2]. Different from the basic and common proteinogenic amino acids (PAAs), which can be found in proteins and have standard genetic codes, non-proteinogenic amino acids (NPAAs) are not naturally encoded, but essential building blocks, because not only do they take part in the composition of peptides and proteins with versatile functions [3,4], they also act as multifunctional molecules, such as drugs (Figure 1a) [5], biotechnological tools (Figure 1b) [6], and catalysts (Figure 1c) [7]. Thus, the construction of NPAAs has continuously attracted attention from chemists and biologists [8]. Compared with the de novo synthesis of NPAAs through enantioselectively constructing bonds around the Cα center [9,10,11], the direct modification of PAAs has been considered as an efficient and convenient strategy to achieve NPAAs rapidly for the ready availability of PAAs. However, the conventional modification of NPAAs relied on the simple organic transformations of highly active groups such as the hydroxyl groups of serine, threonine, and tyrosine, amino groups of arginine and lysine, carboxyl groups of aspartic acid and glutamic acid, as well as the sulfhydryl group of cysteine [12,13,14,15,16]. For the other low active reaction sites, especially the abundant but inert C–H bonds of the PAAs, direct C–H functionalization in modern chemistry has emerged as a powerful way to synthesize versatile NPAAs directly and rapidly [17,18]. Although the transition-metal-catalyzed C–H functionalization of PAAs has been well developed, the employment of expensive and polluting heavy metal, the harsh reaction conditions such as the high temperature limited its practical application to a certain extent. Thus, the development of an alternative method to construct NPAAs directly from PAAs under mild and environmentally friendly conditions is highly desirable considering the deteriorating environmental problems.

**Scheme 1:**
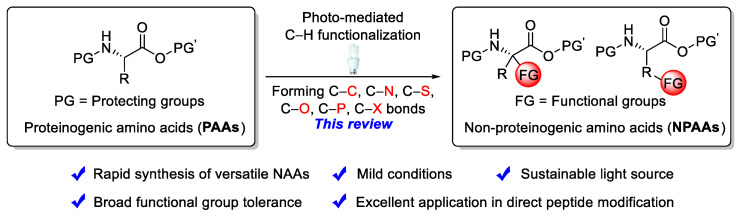
Photo-mediated C–H functionalization of proteinogenic amino acids (PAAs) toward the rapid synthesis of NPAAs.

In recent years, photocatalysis has become an appealing noncanonical synthetic method, in redox [19,20], cross-coupling [21,22], and C–H functionalization reactions [23], due to its mild reaction conditions, sustainable light source, simple operation, as well as the less pollution and waste. Therefore, it is highly attractive to modify PAAs and peptides via photo-mediated C–H functionalization at ambient temperature, exhibiting high competence, broad functional group tolerance, and great energy-saving performance. Although the relevant topic of photocatalytic transformations of amino acids and peptides has been reviewed, it mainly focused on the decarboxylative coupling reactions and functionalization of active sulfydryl groups [24]. In this review, we focus our attention on the recent advances in the photo-mediated C–H functionalization of PAA derivatives directly toward NPAAs (Scheme 1). The reactions are classified according to what kind of chemical bonds are formed (C–C, C–N, C–S, C–O, C–P, C–X bonds) after the photo-mediated C–H activation, and then, the classifications are divided into corresponding C(sp^3^)–H and C(sp^2^)–H activations, respectively.

## 2. C–C Formation

### 2.1. C–H Alkylation

The alkyl group is one of the most fundamental groups. Thus, the development of ideal photo-mediated C–H alkylation reactions has attracted great interest from chemists for their broad applications [25,26].

#### 2.1.1. C(sp^3^)–H Alkylation

**Scheme 2:**
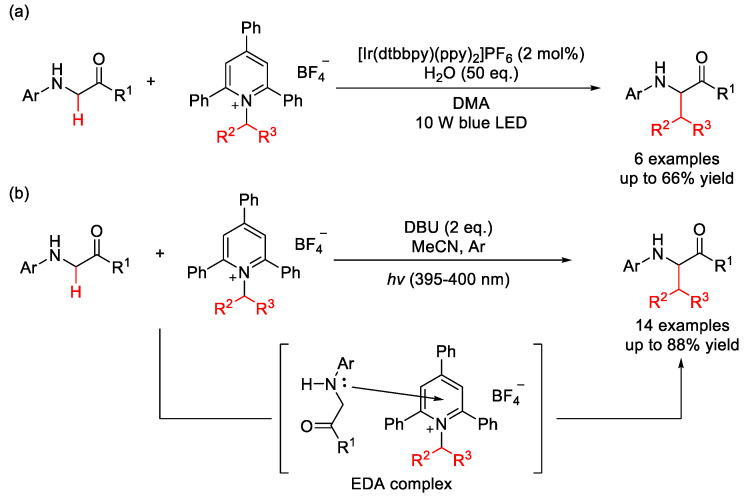
C(sp^3^)–H alkylation of Gly derivatives with *N*-alkyl-2,4,6-triphenylpyridinium salt (TPP) by the catalysis of iridium complex (**a**) and electron donor–acceptor (EDA) complex (**b**).

**Scheme 3:**
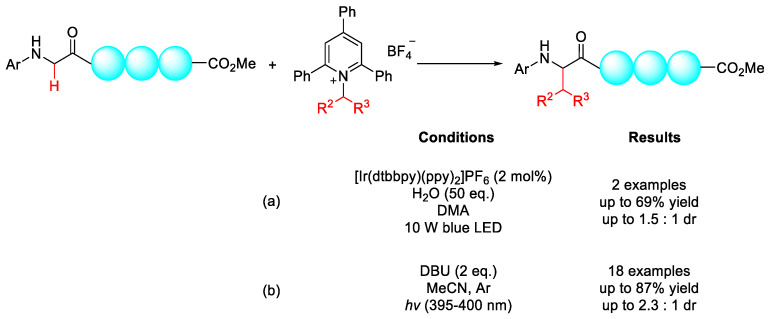
C(sp^3^)–H alkylation of Gly-containing peptides with TPP by the catalysis of iridium complex (**a**) and EDA complex (**b**).

Alkyl Katritzky salts (*N*-alkyl-2,4,6-triphenylpyridinium salts, TPPs) were employed in the photo-mediated C(sp^3^)–H alkylation reactions of glycine derivatives by Lou and Liu’s group [27] and Xu’s group [28], respectively. In Lou and Liu’s work, the authors used [Ir(dtbbpy)(ppy)_2_]PF_6_ as the photocatalyst to excite the Katritzky salts to generate the alkyl radical group to access the alkylated glycine derivatives in moderate efficiency under visible-light irradiation (Scheme 2a). Interestingly, Xu’s group then reported a visible-light promoted catalyst-free C(sp^3^)–H alkylation reaction of glycine derivatives with Katritzky salts in good yields. The electron donor–acceptor (EDA) complex, which was formed with glycine derivatives, Katritzky salts, and the base 1,8-diazabicyclo[5.4.0]undec-7-ene (DBU), could enable the single electron transfer (SET) and subsequent radical coupling to furnish the amino acids functionalization reactions in the presence of the visible light (Scheme 2b). Moreover, these two works both extended the method to the photo-mediated functionalization of glycine-containing peptides, well exhibiting excellent functional group tolerance (Scheme 3).

**Scheme 4:**
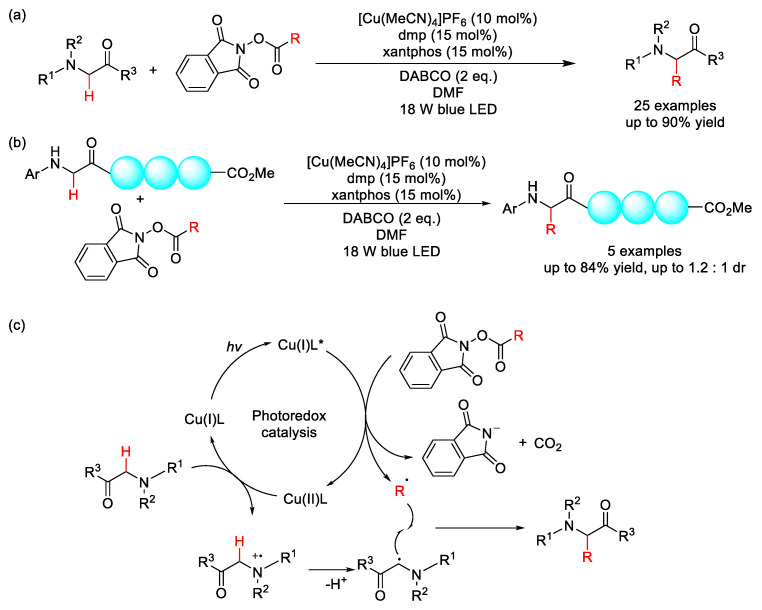
C(sp^3^)–H alkylation of Gly derivatives (**a**) and Gly-containing peptides (**b**) with *N*-hydroxyphthalimide (NHP) esters. (**c**) Proposed mechanism.

*N*-hydroxyphthalimide (NHP) esters, considered as the alternative alkyl precursors, could also allow the photo-mediated alkylation reactions of glycine derivatives (Scheme 4a) and peptides (Scheme 4b) in high yields, as reported by Xu and Wang’s group [29]. The NHP esters could generate alkyl radical in situ through the visible-light-induced Cu-catalyzed decarboxylative process to couple with the glycine derivative carbon radical to accomplish the C(sp^3^)–alkylation reaction of glycine derivatives (Scheme 4c).

**Scheme 5:**
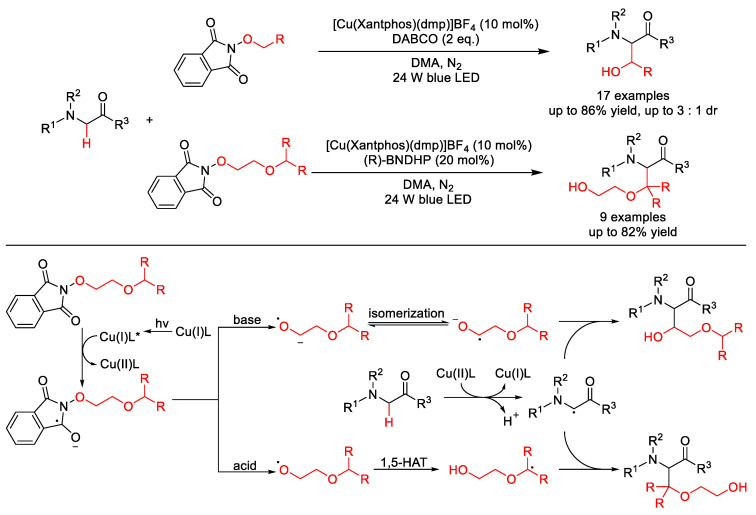
C(sp^3^)–H alkylation of Gly derivatives with *N*-alkoxyphthalimides.

Later in 2019, Duan and Wang’s group used *N*-alkoxyphthalimides as the alkylation reagents in the visible-light-induced Cu-catalyzed C(sp^3^)–alkylation reaction of glycine derivatives (Scheme 5) [30]. Interestingly, the authors have realized the regioselective α- and δ-activation of *N*-alkoxyphthalimide through adding different additives with acidic or alkaline properties. In the presence of basic 1,4-diazabicyclo[2.2.2]octane (DABCO), the reaction exhibited α-selectivity after the resonance to more stable α-oxy carbon-centered radical anion, while in the presence of acidic (*R*)-BNDHP, the generated alkoxy radical after protonation would subsequently undergo 1,5-HAT to give the remote carbon-centered radical. Then, these two kinds of radicals coupled with glycine radical cation, which was produced along with the regeneration of Cu(I) to yield the final α- and δ-selective product. It is worth noting that *N*-alkoxyphthalimides show different chemical activity to generate alkoxy radicals first rather than the direct generation of the alkyl radicals compared with the NHP esters.

**Scheme 6:**
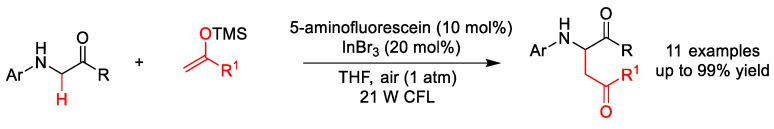
C(sp^3^)–H alkylation of Gly derivatives with silyl enol ethers.

In 2017, Sugiyama and Park’s group has developed a photo-induced alkylation of glycine derivatives with silyl enol ethers as another kind of alkylation reagent (Scheme 6) [31]. This reaction was cooperatively catalyzed by 5-aminofluorescein and InBr_3_. 5-Aminofluorescein as the photocatalyst was excited to oxidize the glycine derivatives to imines, while InBr_3_ was added to facilitate the Mannich-type reaction of imines and silyl enol ethers.

**Scheme 7:**
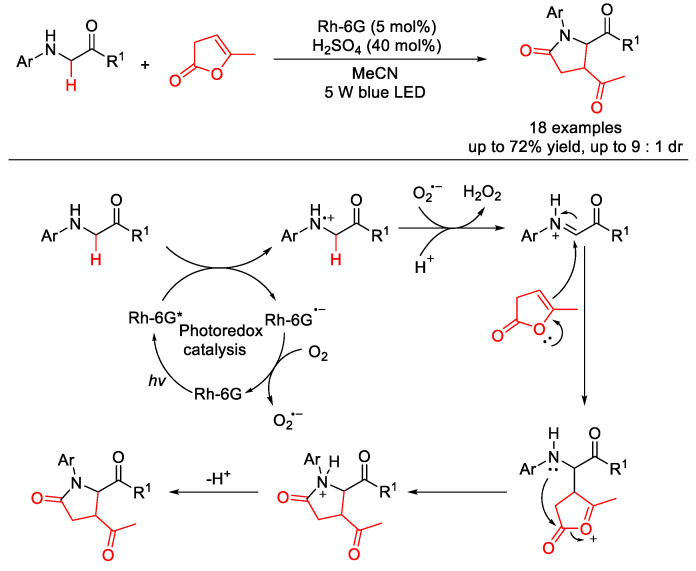
C(sp^3^)–H alkylation of Gly derivatives with α-angelicalactone.

Apart from silyl enol ethers, enol ester like α-angelicalactone was employed by Zhang and Li’s group as the alkylation reagent to furnish the tandem C(sp^3^)–alkylation and intramolecular cyclization reaction of glycine derivatives under the merging catalysis of Rh-6G and H_2_SO_4_ to form the γ-lactams (Scheme 7) [32]. The acid catalysis is critical to this transformation, because only a trace amount of product was observed in the control experiments without H_2_SO_4_.

**Scheme 8:**
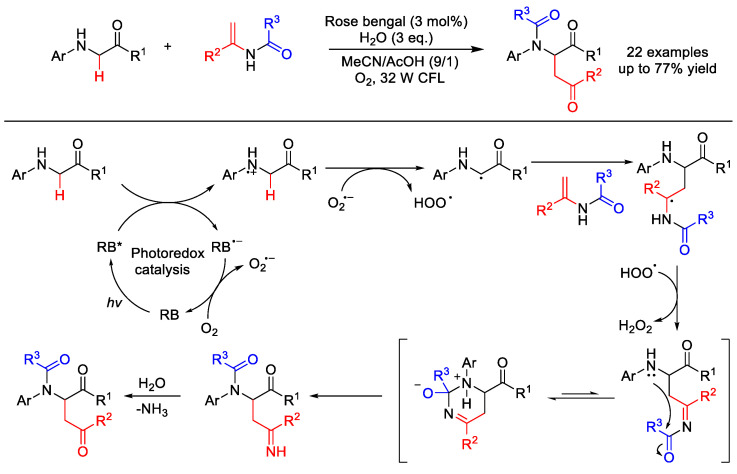
C(sp^3^)–H alkylation of Gly derivatives with enamines.

Guan and He’s group also attempted the *N*-acetylenamine as the similar alkylation reagent in the Rose Bengal-catalyzed photo-oxidative alkylation of glycine derivatives (Scheme 8) [33]. Similar to the above-mentioned enols, here, the *N*-acetylenamines captured the carbon radicals of glycine derivatives to generate carbon radical intermediates, which were abstracted as a hydrogen atom and then underwent cyclization, rearrangement, and hydrolysis to obtain the final *N*-acylation-α-alkylation products in good yields.

**Scheme 9:**
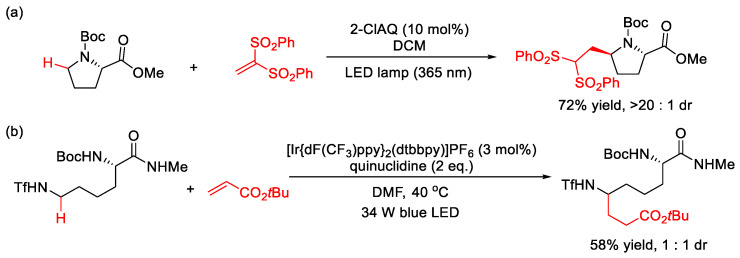
C(sp^3^)–H alkylation of amino acids derivatives with 1,1-bis(phenylsulfonyl)ethylene (**a**) and *tert*-butyl acrylate (**b**).

Electron-deficient olefins were also investigated as alkylation reagents employed in the photo-mediated C(sp^3^)–alkylation of amino acids. In 2016, Kamijo’s group described the 2-ClAQ-catalyzed alkylation of proline derivate with 1,1-bis(phenylsulfonyl)ethylene in 72% yield (Scheme 9a) [34]. Additionally, the installed bis(phenylsulfonyl)ethylene group was a helpful synthetic intermediate for further versatile transformations. Later in 2019, Rovis’s group also reported an example of photo-mediated functionalization of the lysine derivative with *tert*-butyl acrylate in 58% yield with 1:1 dr value (Scheme 9b) [35]. Notably, the authors have tested other protecting groups such as the trifluoroacetyl group, *p*-toluenesulfonyl group, and acetyl group, and they are all deleterious to this process, which indicated that the trifluoromethanesulfonyl group as the protecting group on the nitrogen atom was extremely essential to the reaction.

**Scheme 10:**
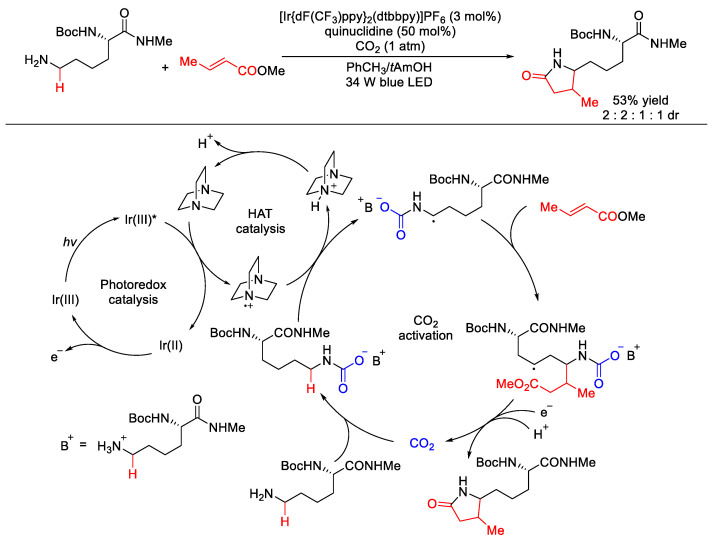
CO_2_-activated tandem C(sp^3^)–H alkylation and intramolecular cyclization.

As it remains challenging to functionalize C(sp^3^)–H of the substrates containing unprotected primary amines, Rovis and Schoenebeck’s group has developed a CO_2_-activated tandem C(sp^3^)–H alkylation and intramolecular cyclization to deliver the γ-lactam derivative of lysine (Scheme 10) [36]. CO_2_ first activated the primary amine to form the alkylammonium carbamate, which would be abstracted as a hydrogen atom to generate the carbon-centered radical to furnish the radical coupling and dissociate the CO_2_ to give the final product. According to the experimental and computational results, CO_2_ could act as a protecting group to prevent the *N*-alkylation and a negatively charged carbamate anion to simultaneously accelerate the intermolecular hydrogen atom transfer (HAT) process. 

**Scheme 11:**
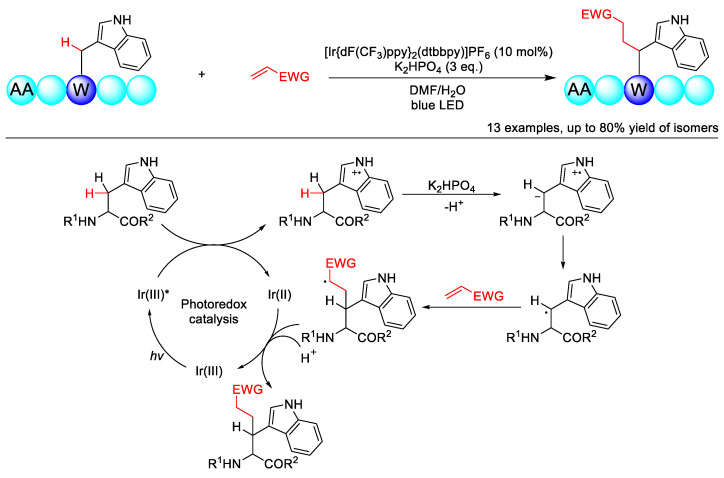
C(sp^3^)–H alkylation of Trp-containing peptides with electron-deficient olefins.

Shi and coworkers described an example of the photo-mediated C(sp^3^)–H alkylation reaction of the β-position of the tryptophan-containing peptides with high levels of selectivity (Scheme 11) [37]. The reaction exhibited a broad substrate scope for tolerating several endogenous peptides and electron-deficient olefins. Interestingly, only tryptophan could proceed in the process, while other similar benzyl-containing residues such as phenylalanine and tyrosine had no reaction. The possible highly selective mechanism might be that the benzylic proton was more acidic than others due to the formation of the indole radical cation after the one-electron oxidation of indole *N* atom. So, the β-H could be abstracted by the base, and the more stable carbon radical was formed to couple with the electron-deficient olefins to realize the photo-mediated C(sp^3^)–H alkylation reaction of the tryptophan-containing peptides.

**Scheme 12:**
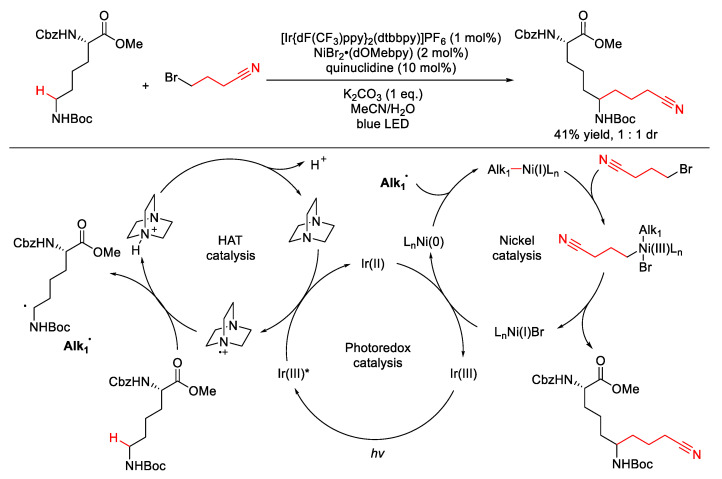
C(sp^3^)–H alkylation of Lys derivatives with alkyl bromides.

**Scheme 13:**
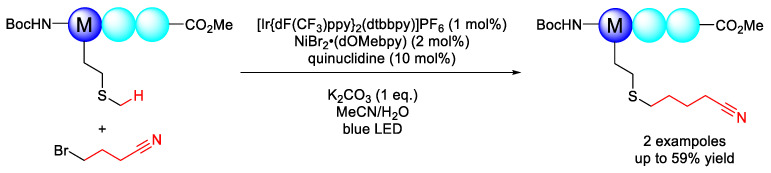
C(sp^3^)–H alkylation of Met-containing peptides with alkyl bromides.

In 2017, MacMillan’s group has described a C(sp^3^)–alkylation of lysine derivatives using alkyl bromide as the alkylation reagent (Scheme 12) [38]. The butyronitrile group has been functionalized at the position adjacent to the nitrogen atom of lysine derivative under the irradiation of visible light. This alkylation process involved three catalysis (HAT catalysis, photoredox catalysis, and nickel catalysis) simultaneously. The proposed mechanism of the triple catalysis is as follows. First, the photocatalyst was excited under the irradiation of the visible light to form the excited state, which then oxidizes quinuclidine to give the cationic radical. The cationic radical and the lysine derivative engaged in the HAT catalytic cycle to form the alkyl radical of the lysine derivative (**Alk_1_·**), which is trapped by Ni(0) to arrive at the Ni(I)-alkyl intermediate. Oxidative addition with 4-bromobutanenitrile and subsequent reductive elimination obtained the final alkylation product, along with the Ni(I) complex, which could be reduced by Ir(II) to regenerate the Ni(0) catalyst for the next nickel catalytic cycle. Additionally, this method has also been applied to the late-stage functionalization of peptides containing methionine residues in moderate yields with high selectivity (Scheme 13).

**Scheme 14:**
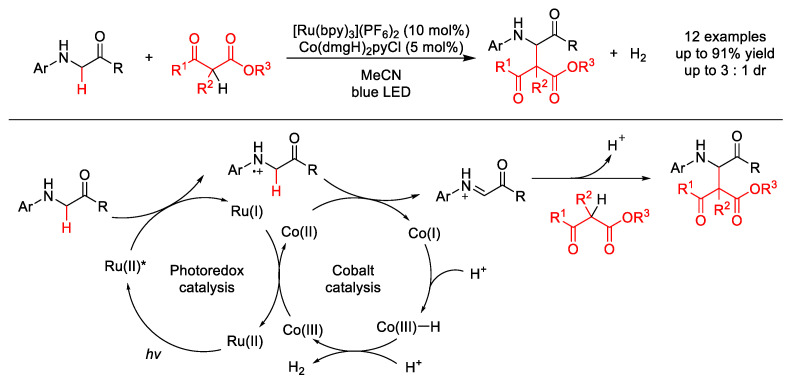
C(sp^3^)–H alkylation of Gly derivatives with the β-keto esters.

What is more, Wu’s group has described a C(sp^3^)–H functionalization reaction of glycine esters with the β-keto esters in 2015, which was cooperatively catalyzed by [Ru(bpy)_3_](PF_6_)_2_ and Co(dmgH)_2_pyCl (Scheme 14) [39]. In this case, β-keto esters containing reactive α-Hs as alkylation reagents were used to produce the alkylated glycine derivatives. Significantly, this cross-coupling hydrogen evolution reaction avoided the requirement of equivalent amounts of bases or oxidants to activate the β-keto esters and generated only H_2_ as the by-product, because the cobalt catalyst was chosen to capture the electrons and protons eliminated from the β-keto esters, which made this method economical and environmentally benign.

#### 2.1.2. C(sp^2^)–H Alkylation

**Scheme 15:**
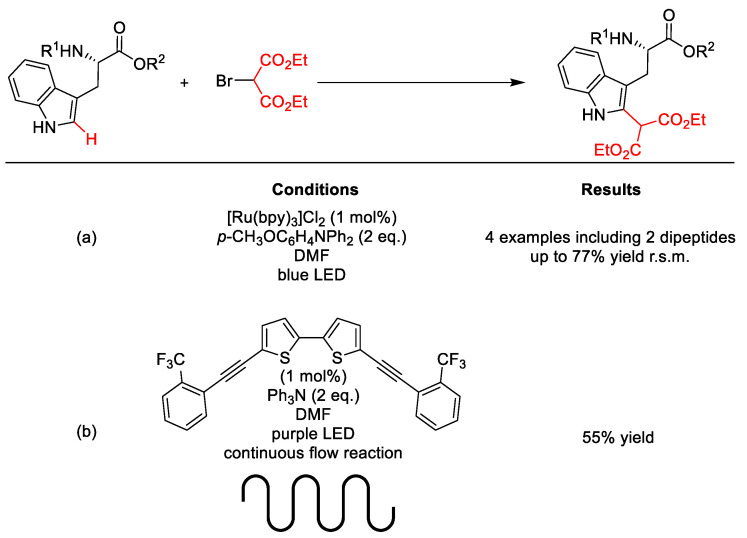
C(sp^2^)–H alkylation of Trp derivatives with alkyl bromides by the catalysis of ruthenium complex (**a**) and bithiophene (**b**).

In 2010, the C(sp^2^)–alkylation of tryptophans has been realized by Stephenson’s group with diethyl bromomalonate under the catalysis of [Ru(bpy)_3_]Cl_2_ (Scheme 15a) [40]. Additionally, dipeptides were also compatible in this alkylation reaction. Later in 2019, Noël and Carrillo’s group has developed a novel 2,2′-bithiophene-based organic photocatalyst for photoredox C–H alkylation in a continuous-flow photo-microreactor (Scheme 15b) [41].

**Scheme 16:**
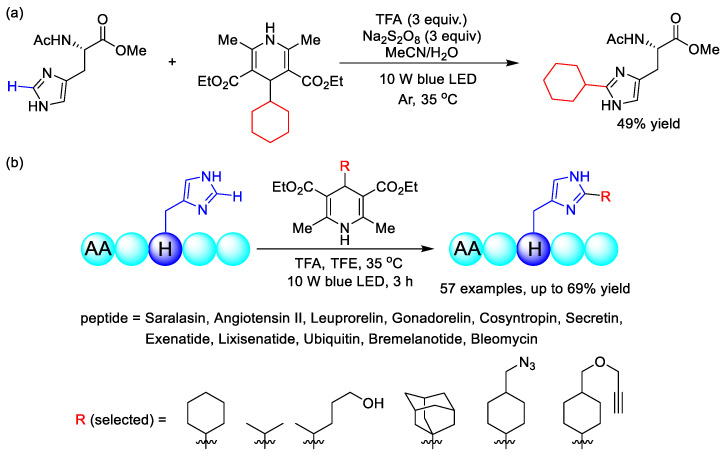
C(sp^2^)–H alkylation of His derivatives (**a**) and His-containing peptides (**b**) with 4-alkyl-1,4-dihydropyridines (DHP).

4-Alkyl-1,4-dihydropyridine (DHP) reagents were also employed as alkylation reagents in the C(sp^2^)–alkylation of histidine derivatives with the assistance of the oxidant Na_2_S_2_O_8_ by Wang and Chen’s group in 2019 (Scheme 16a) [42]. Various alkyl groups containing reactive functional groups (such as the hydroxy group, azido group, and the alkynyl group, etc.) could be installed on bioactive peptides, peptide drugs, and even a small protein in moderate to good yields and excellent selectivities when omitting Na_2_S_2_O_8_ (Scheme 16b). Notably, the avoidance of the strong external oxidant could suppress the formation of undesired by-products, because the peptide containing Tyr, Trp, and Met residues would be oxidized by the strong oxidants, which was a serious problem in the modification of peptides.

**Scheme 17:**
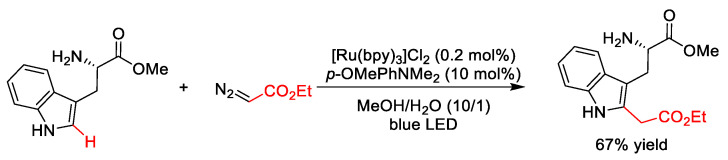
C(sp^2^)–H alkylation of Trp derivatives with diazo esters.

In addition, diazo compounds are a kind of alternative reactive chemical reagent that are commonly used in C(sp^2^)–alkylation reactions. Gryko’s group has described the photoalkylation of tryptophans with diazo esters in 67% yield (Scheme 17) [43]. It was worth noting that the catalyst loading could be as low as 0.2% when the reaction scale was 0.25 mmol, which is beneficial to scale up and apply in industry.

**Scheme 18:**
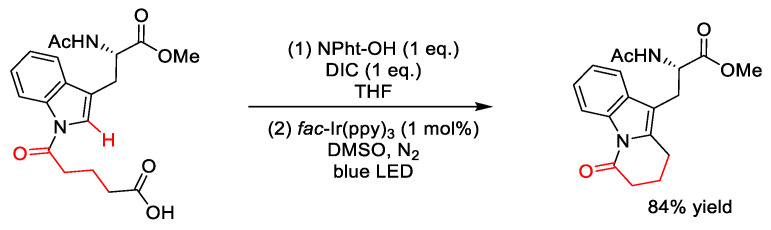
Intramolecular decarboxylative alkylation of Trp derivatives.

In addition to intermolecular alkylation reactions, the intramolecular photocatalyzed alkylation of tryptophan was also investigated when tryptophan was substituted by a glutaroyl group (Scheme 18) [44]. The carboxylic acid was first coupled with NPht-OH and then underwent photocatalytic decarboxylative alkylation, leading to the pyridotryptophan through a one-pot protocol. The authors hope to use this methodology in the rapid synthesis of versatile indole-containing bioactive molecules and natural products.

### 2.2. C–H Benzylation

C–H benzylation is an attractive method to introduce the benzyl group to construct the bioactive structures [45]. Especially, the important diarylmethanes compounds could be achieved through the C(sp^2^)–H benzylation of the (hetero)arenes [46].

#### 2.2.1. C(sp^3^)–H Benzylation

**Scheme 19:**
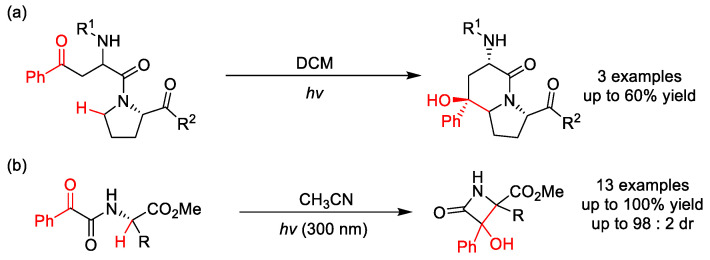
Intramolecular C(sp^3^)–H benzylation of proline derivatives (**a**) and other amino acids derivatives (**b**) with phenyl ketones.

Wessig’s group [47] and Griesbeck’s group [48] have independently reported the catalyst-free photo-mediated intramolecular C(sp^3^)–benzylation of amino acids derivatives in 1999 and 2002, respectively. In Wessig’s work, dipeptides of l-β-benzoylalanine and proline esters could deliver the bicyclic indolizinones under the photoirradiation (Scheme 19a). Notably, the protecting group R^1^ could be Cbz, Alloc, and Boc, rather than Fmoc, because the biphenyl skeleton of Fmoc would quench the triplet excited benzoyl group to suppress this process. In Griesbeck’s work, β-lactams were produced from phenylglyoxyl amino acid derivatives in excellent yields with broad substrate scope and good to high diastereoselectivities (Scheme 19b). Unfortunately, this transformation had no memory of chirality to induce the asymmetric cyclization.

**Scheme 20:**
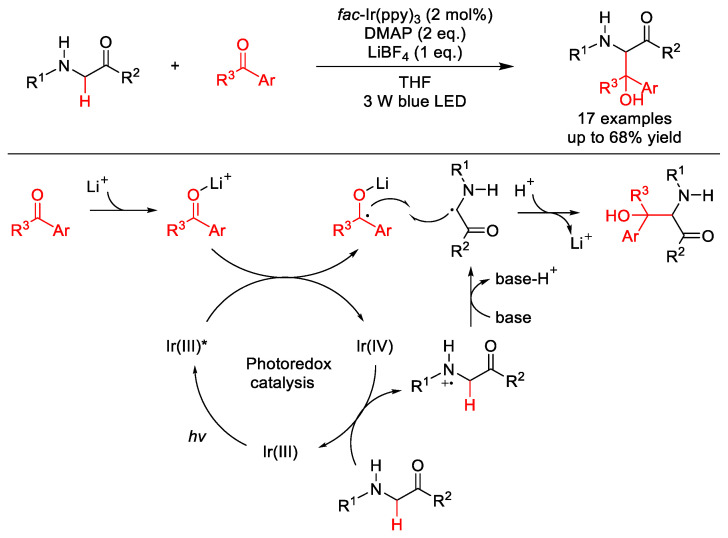
Intermolecular C(sp^3^)–H benzylation of Gly derivatives with phenyl ketones.

**Scheme 21:**
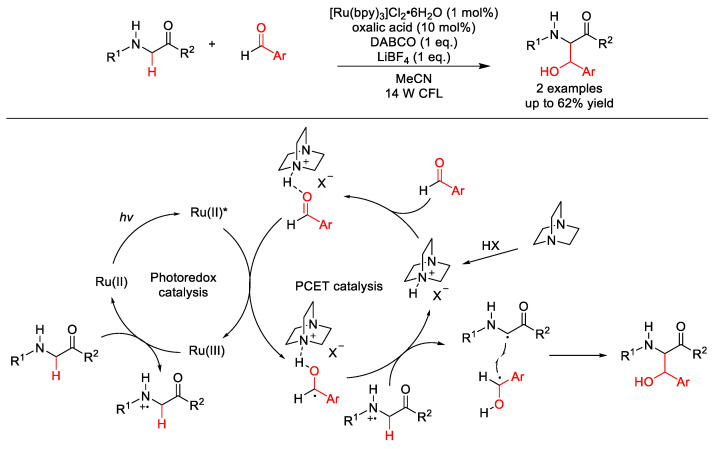
Intermolecular C(sp^3^)–H benzylation of Gly derivatives with aldehydes.

Since 2016, Xiao’s group [49] and Wang’s group [50] have developed the photocatalytic benzylation of glycine derivatives with ketones and aldehydes, respectively, to produce useful 1,2-amino alcohols in moderate efficiency. Xiao and coworkers believed that the lithium salt LiBF_4_ took the important role as a Lewis acid in the activation of the ketones to the corresponding ketyl radical (Scheme 20). Then, the ketyl radical coupled with the amino radical to form the final product after a protonation process. While in Wang’s work, the authors proposed a proton-coupled electron transfer (PCET) pathway of the complex containing the aldehyde, DABCO, and a protonic acid (Scheme 21).

**Scheme 22:**
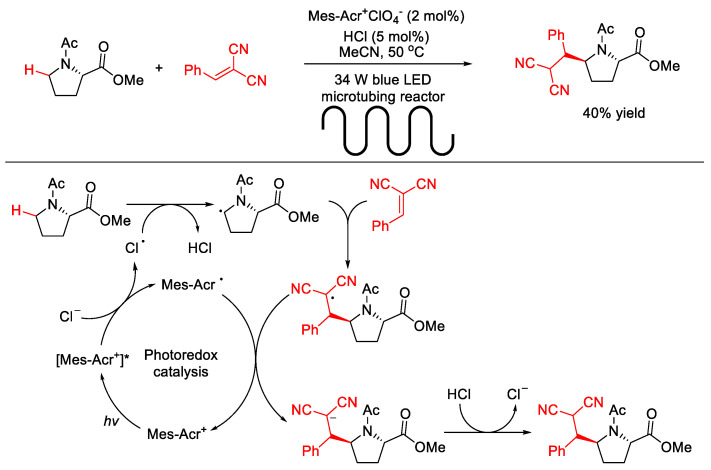
C(sp^3^)–H benzylation of Pro derivative with benzylidenemalononitrile.

Apart from the intra- or intermolecular carbonyl groups, benzylidenemalononitrile could also be considered as an alternative photo-benzylation reagent. It was employed in the benzylation reaction of proline derivative catalyzed by the acridinium photocatalyst Mes-Acr^+^ClO_4_^−^ under the irradiation of 34 W blue light emitting diode (LED) (Scheme 22) [51]. Notably, the stop-flow microtubing (SFMT) reactor showed better results than the conventional batch reactor. Moreover, the catalytic amount of HCl was essential to the photocatalytic process for its function in hydrogen atom transfer (HAT) to activate C(sp^3^)–H.

#### 2.2.2. C(sp^2^)–H Benzylation

**Scheme 23:**
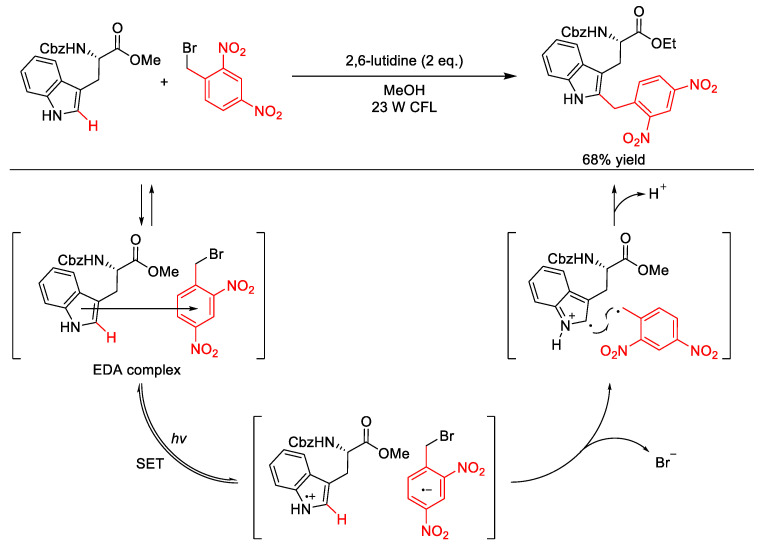
Catalyst-free C(sp^2^)–H benzylation of Trp derivatives with benzyl bromide through the electron donor–acceptor (EDA) complex.

In 2015, a metal-free, photochemical strategy for the C–H benzylation of tryptophan has been reported by Melchiorre’s group (Scheme 23) [52]. The benzylated product was received in 68% yield through the photochemical activity of the EDA complex from tryptophan and benzyl bromides with electron-withdrawing groups, such as 2,4-dinitrobenzyl bromide. Importantly, the EDA complex has been successfully isolated and unambiguously characterized by X-ray, and it has been proved to have the photochemical activity in this benzylation reaction, which provided valuable information for further investigations on the EDA complex in photochemistry.

**Scheme 24:**
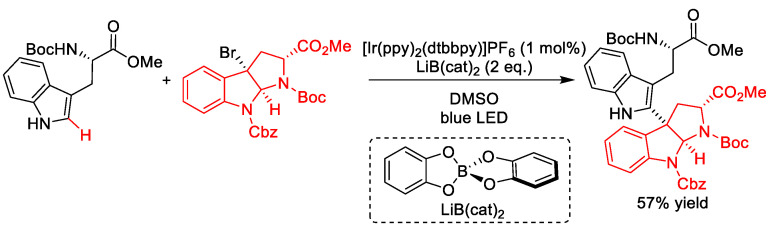
C(sp^2^)–H benzylation of Trp derivatives with tricyclic benzyl bromide.

In 2018, Stephenson’s group has developed lithium bis-catechol borate (LiB(cat)_2_) as a novel, efficient, and inexpensive reductive quencher in the photoredox-mediated C–H benzylation due to its absence of α-protons to avoid undesired HAT, which commonly occurred when trialkyl amines were employed as sacrificial reductive quenchers (Scheme 24) [53]. The redox potential of LiB(cat)_2_ was similar to other candidates, while the cost was significantly lower than the others, such as the commercially available 4-methoxy-*N*,*N*-diphenylaniline. Therefore, the authors employed LiB(cat)_2_ as a reductive quencher to furnish the functionalization of tryptophan with pyrroloindolines.

### 2.3. C–H Allylation

**Scheme 25:**
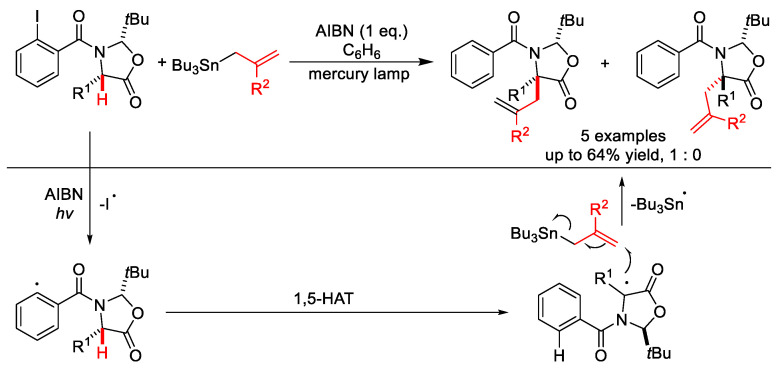
C(sp^3^)–H allylation of amino acid derivatives with allyltributyltin.

Wood’s group has realized an intermolecular photo-accelerated α-allylation of *N*-(2-iodobenzoyl) amino acid derivatives in 2009 (Scheme 25) [54]. With the increase of the steric hindrance of R^1^ (R^1^ = H, R^1^ = Me, R^1^ = *i*Pr), the yield of the corresponding product would dramatically decline, indicating the difficulty in constructing remarkably hindered quaternary carbon centers. Mechanically, the allylation process might involve the aryl radical formation, hydrogen transfer to generate the key α-aminoalkyl radical intermediate, allyl radical formation, and radical coupling processes to finish the allylation reaction.

### 2.4. C–H Fluoroalkylation

Due to the important role of fluoroalkyl group in biomolecules to increase the reactivity, solubility, and polarity, fluoroalkylation particularly attracted researchers’ interests [55,56,57].

**Scheme 26:**
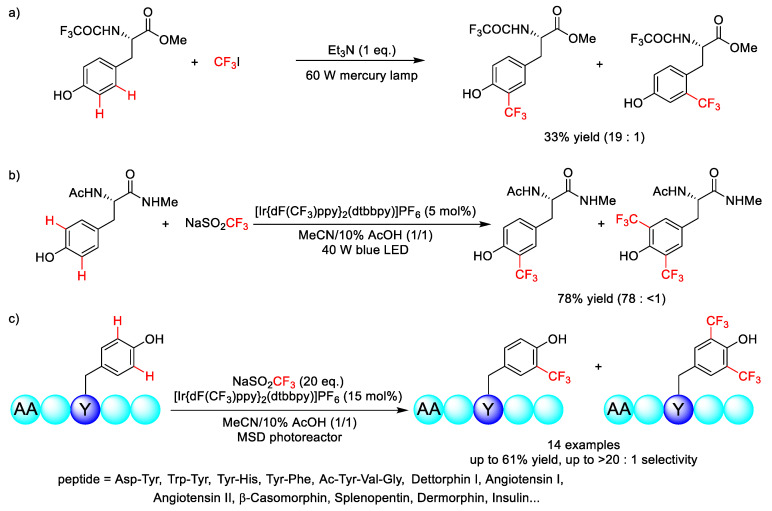
C(sp^2^)–H trifluoromethylation of Tyr derivatives with CF_3_I (**a**) and NaSO_2_CF_3_, (**b**) Tyr-containing peptides with NaSO_2_CF_3_ (**c**).

Early in 1992, Kimoto’s group found that the photo-irradiation of a mixture of tyrosine and CF_3_I could lead to the trifluoromethylation of the tyrosine derivative in 33% yield (Scheme 26a) [58]. Apart from the *ortho*-trifluoromethylation, a small amount of *meta*-trifluoromethylation product was also found according to the ^19^F NMR and GC-MS. As a result of the gaseous property and the inconvenience of using CF_3_I, NaSO_2_CF_3_ has been developed as an alternative trifluoromethylation reagent in the visible-light-mediated trifluoromethylation of tyrosine and its peptides by Krska and Parish’s group in 2018 (Scheme 26b) [59]. Interestingly, different from Kimoto’s work, bis-CF_3_ tyrosine as another kind of side product was generated under the conditions. What is more, 14 examples of peptides including bioactive angiotensin, casomorphin, dermorphin, and even insulin could well participate in the trifluoromethylation reactions (Scheme 26c).

**Scheme 27:**
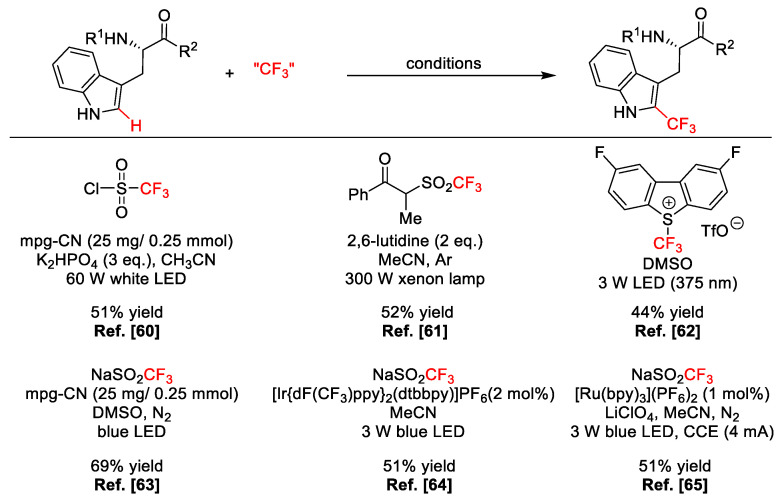
C(sp^2^)–H trifluoromethylation of Trp derivatives.

Trifluoromethylation of tryptophans from plentiful trifluoromethyl synthons in photochemistry has been widely investigated (Scheme 27). Trifluoromethanesulfonyl chloride, 2-(trifluoromethylsulfonyl)ethanone, and Umemoto reagent II have been used in the trifluoromethylation reactions independently by Blechert’s group [60], Li’s group [61], Hamashima and Egami’s group [62], since 2015. NaSO_2_CF_3_ as a stable, inexpensive, and easy-handling trifluoromethyl synthon has been applied in the trifluoromethylation of tryptophans catalyzed by the mesoporous graphitic carbon nitride (mpg-CN) [63] and iridium complex [64], respectively. Notably, electrochemistry was merged with photochemistry to catalyze the trifluoromethylation of tryptophan with NaSO_2_CF_3_ [65]. The cooperative catalysis by electrophotochemistry relied on the anodic electrooxidation to undergo the regeneration of the photocatalyst, rather than the chemical oxidants.

**Scheme 28:**
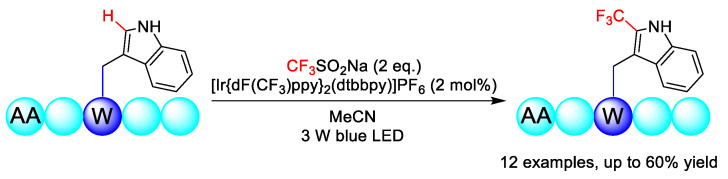
C(sp^2^)–H trifluoromethylation of Trp-containing peptides with NaSO_2_CF_3_.

Particularly, Chiang’s group has expended the scope of the visible-light-induced photoredox trifluoromethylation to the tryptophan-containing peptides with excellent chemo- and site-selectivity under mild and biocompatible conditions (Scheme 28) [64].

**Scheme 29:**
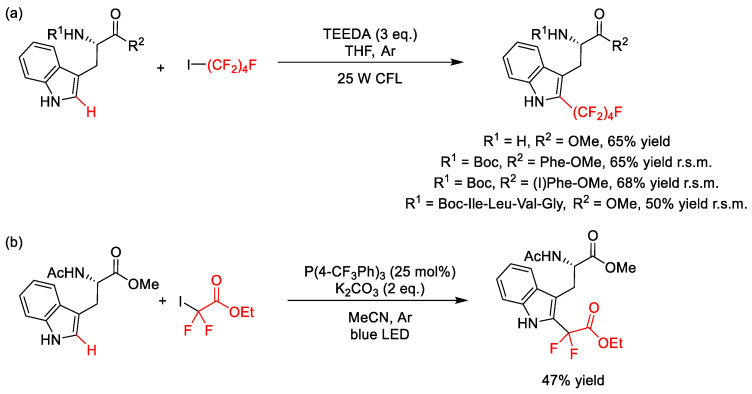
C(sp^2^)–H fluorobutylation (**a**) and *gem*-difluoromethylation (**b**) of Trp derivatives.

Other than the trifluoromethyl group, the perfluorobutyl group was also functionalized on the tryptophan and its peptides in moderate yields under a photocatalyst-free condition reported by Chen’s group (Scheme 29a) [66]. Gem-difluoromythylation was realized using triaryl phosphine as an electron donor to combine with 2,2-difluoro-2-iodoacetate for the formation of an EDA complex facilitating the reaction by Zhang’s group in 2020 (Scheme 29b) [67].

### 2.5. Miscellaneous C–H Alkylation

#### 2.5.1. C(sp^2^)–Phosphonoacetylation

**Scheme 30:**
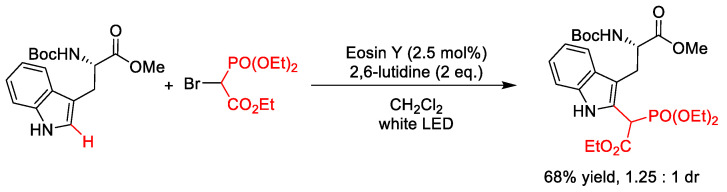
C(sp^2^)–H phosphonoacetylation of Trp derivative with 2-bromophosphonoacetic ester.

A metal-free phosphonoacetylation of tryptophan derivative with 2-bromophosphonoacetic ester under the catalysis of Eosin Y has been described by Opatz’s group in 2018 (Scheme 30) [68]. The synthetic potential of further transformation of phosphonates through Horner-olefinations made this research of great importance and application value.

#### 2.5.2. C(sp^2^)–Diazomethylation

**Scheme 31:**
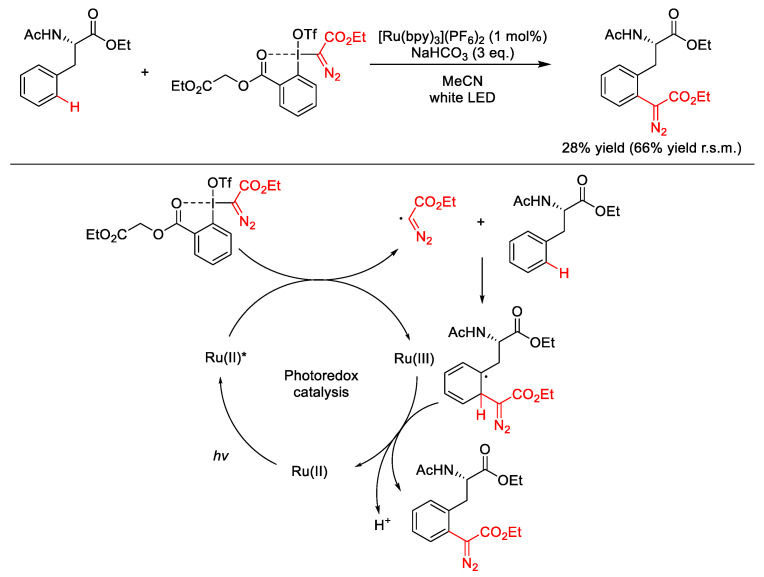
C(sp^2^)–H diazomethylation of Phe derivative with benziodoxolone.

Carbynes as fundamental and highly electrophilic but arcane intermediates have been largely unexplored, resulting from the limited carbyne equivalents and generation strategies [69,70]. Hence, investigations on carbynes and their equivalents are highly desirable to help chemists better understand chemical reactivities. Suero’s group described a photocatalyzed diazomethylation of phenylalanine with benziodoxolone as a carbyne equivalent species in 2018, which provided a great example for carbyne’s generation and subsequent transformation (Scheme 31) [71].

### 2.6. C–H Alkenylation

The alkenyl group is an extraordinary powerful structure, which could be further transformed through the addition, oxidation, reduction, annulation and transition-metal-catalyzed cross-coupling reaction, such as a Heck reaction [72]. Therefore, the introduction of the alkenyl group into the amino acids has also been investigated for the remarkably increasing potential diversity of the products and also the intermediates in the total synthesis of natural products [73].

**Scheme 32:**
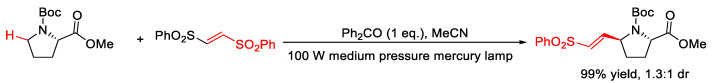
C(sp^3^)–H alkenylation of Pro derivative with bis(phenylsulfonyl)ethylene.

In 2014, Inoue and Kamijo’s group reported a metal-free sulfonylalkenylation reaction of proline derivative with bis(phenylsulfonyl)ethylene in quantitive yield under the photo-irradiation conditions (Scheme 32) [74]. The sulfonylalkene could be easily converted to prenyl alcohol and pyrrole derivative, which gives this transformation possess extended synthetic utility.

**Scheme 33:**
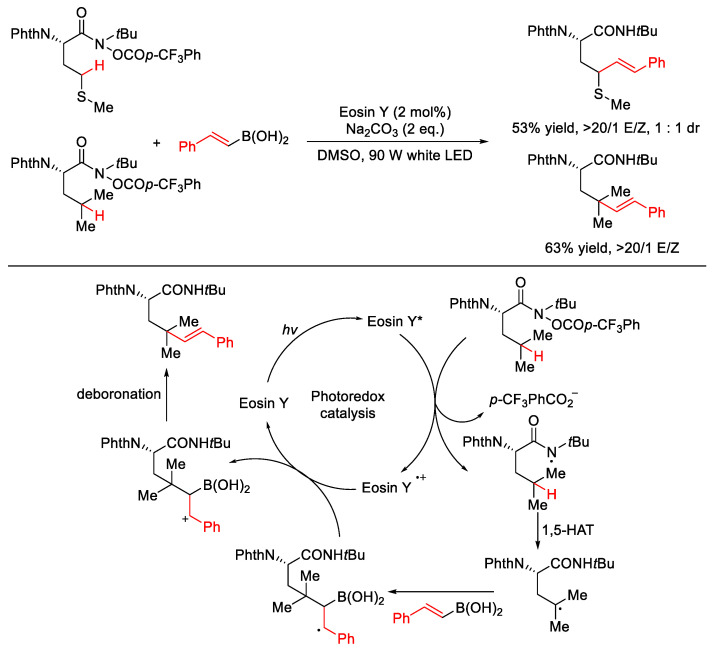
C(sp^3^)–H alkenylation of Met and Leu derivatives with alkenylboronic acid.

Alkenylboronic acids have also been developed as visible-light-mediated alkenylation reagents for the styrenation of methionine and leucine catalyzed by Eosin Y under mild conditions (Scheme 33) [75]. Taking the leucine for example, an amidyl radical is formed by single-electron transfer from Eosin Y*, generating the carboxylate anion (*p*-CF_3_PhCO_2_^−^) at the same time. Subsequently, the C(sp^3^)–H bond would be activated through 1,5-HAT to give the carbon-centered radical. Then, it could attack the alkenylboronic acid to undergo SET and deboronation to deliver the final styrenation product.

### 2.7. C–H Alkynylation

**Scheme 34:**
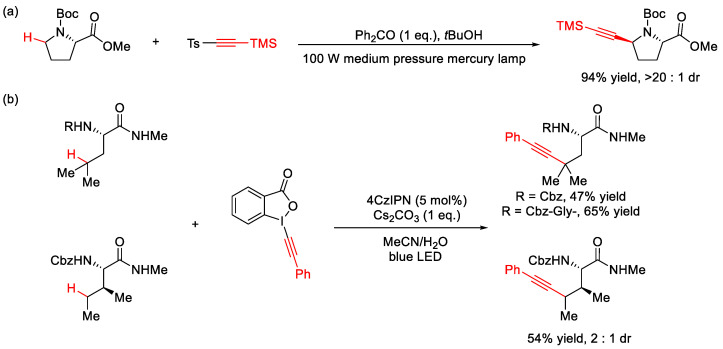
C(sp^3^)–H alkynylation of amino acids derivatives with 1-tosyl-2-(trimethylsilyl)acetylene (**a**) and ethynylbenziodoxolone (**b**).

In 2013, Inoue’s group has developed a photochemical alkynylation of proline with 1-tosyl-2-(trimethylsilyl)acetylene when diphenyl ketone worked as a precursor reagent (Scheme 34a) [76]. Moreover, the alkynylated proline derivative could be easily transformed to corresponding carboxylic acid and phenylacetylene efficiently. Ethynylbenziodoxolone reagents could also incorporate the phenylacetyl group in valine and isoleucine catalyzed by 4CzlPN (Scheme 34b) [77]. A dipeptide (Cbz-Gly-Val-NHMe) was also functionalized smoothly in 65% yield under visible-light irradiation.

### 2.8. C–H Acylation

C–H acylation remains a powerful and convenient strategy to introduce the carbonyl group into the organic compounds to generate the versatile ketones [78,79].

#### 2.8.1. C(sp^3^)–H Acylation

**Scheme 35:**
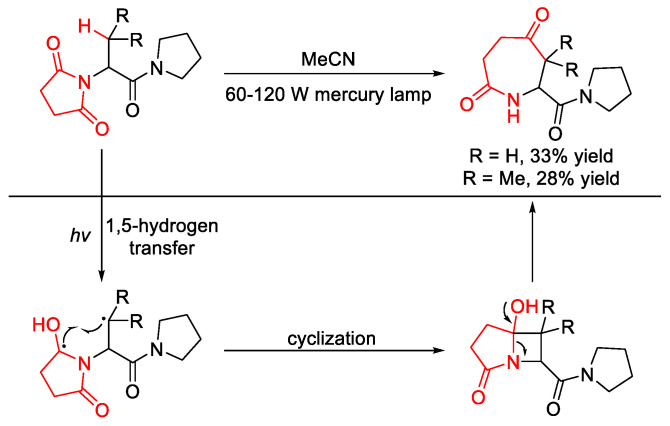
Intramolecular C(sp^3^)–H acylation of Ala and Val derivatives with *N*-succinimides.

An intramolecular photocatalysis C(sp^3^)–H acylation of β-position of alanine (R = H) and valine (R = Me) *N*-succinimides to deliver the medium-ring product under the photoirradiation of a low-pressure mercury lamp was reported in 1986 (Scheme 35) [80]. The succinimide first abstracts a hydrogen atom and then undergoes the cyclization to give an azacyclobutanol intermediate. Due to its high ring strain, a retro-transannular process happens to obtain the final product.

**Scheme 36:**
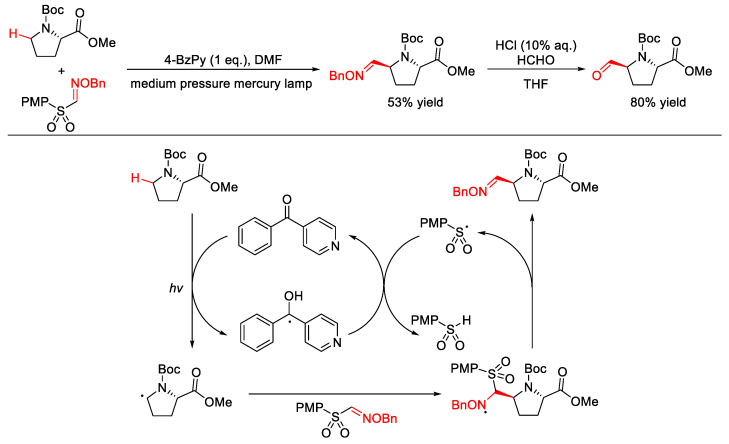
Intermolecular C(sp^3^)–H acylation of Pro derivative with sulfonyl oxime.

Afterwards, an intermolecular C(sp^3^)–acylation of proline was developed by Kamijo’s group in 2016 through a tandem aldoxime group introduction and hydrolysis (Scheme 36) [81]. A proline derivative is abstracted as hydrogen by photoexcited 4-benzoylpyridine (4-BzPy), and it attacked the sulfonyl oxime to provide the aminyl radical. Finally, the product is afforded through the left of the sulfonyl radical. Formylated proline could be easily achieved after the acid treatment with formaldehyde.

#### 2.8.2. C(sp^2^)–H Acylation

**Scheme 37:**
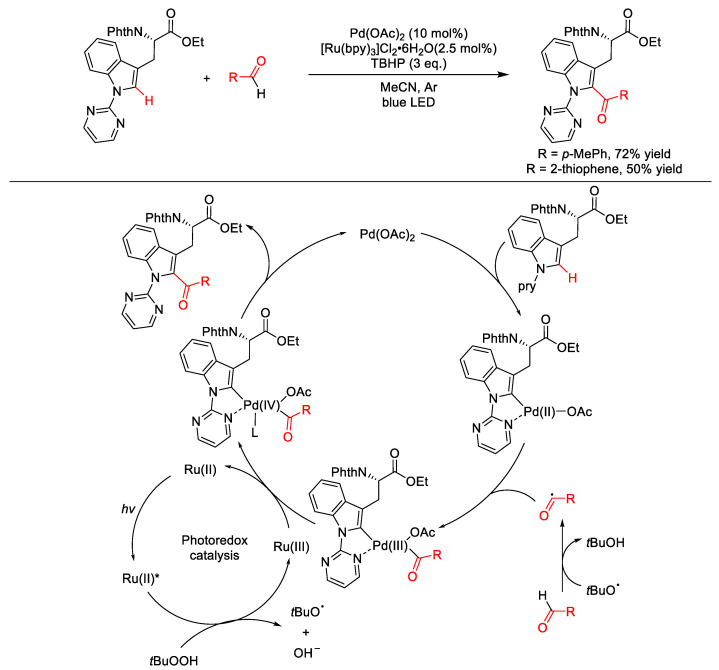
C(sp^2^)–H acylation of Trp derivatives with aldehydes.

The acylation of tryptophan with aldehydes under the cooperative catalysis of visible-light photoredox and palladium with high efficiency has been reported by Jana’s group in 2017 (Scheme 37) [82]. Pyrimidine was chosen as the directing group substituted on the nitrogen of the indole to coordinate palladium for C–H activation. Additionally, *tert*-butyl hydroperoxide (TBHP) was essential in the reaction due to the failure of the reaction without TBHP. Mechanically, the oxidative addition of the palladium complex with the acyl radical, generated from the aldehyde under the photocatalysis, occurred to form the Pd(III) intermediate. Then, the desired product was obtained through single electron transfer and reductive elimination, and finally, the palladium catalyst was regenerated for the next catalytic cycle. Although this work merged the photocatalysis and palladium-catalysis perfectly for the acylation of tryptophan, the introduction of the pyrimidine-directing group increased the difficulty for practical application to some extent.

### 2.9. C–H Cyanation

**Scheme 38:**
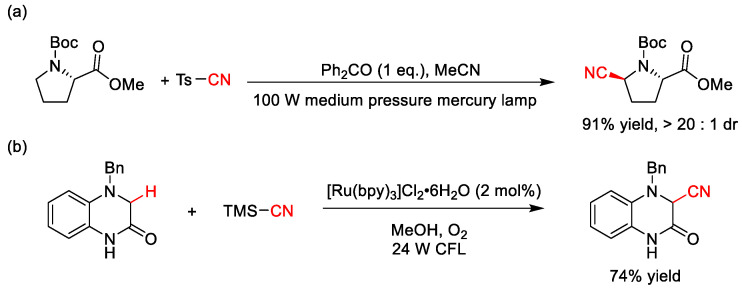
C(sp^3^)–H cyanation of Pro (**a**) and Gly (**b**) derivatives.

In 2011, Inoue’s group has developed a protocol for the direct photo-cyanation of proline with TsCN under the irradiation of a 100 W medium pressure mercury lamp in high efficiency and diastereoselectivity (Scheme 38a) [83]. Additionally, TMSCN was used as an alternative cyanation reagent to provide the cyanated glycine derivative under the visible-light catalysis in 2018 (Scheme 38b) [84].

**Scheme 39:**
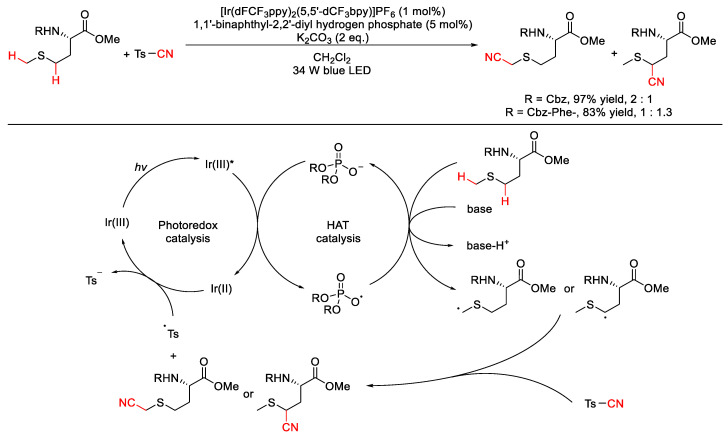
C(sp^3^)–H cyanation of Met derivatives through the cooperative visible-light photoredox/phosphate acid catalysis.

Particularly in 2018, Kanai and Oisaki’s group reported a C(sp^3^)–H cyanation of methionine and its dipeptide through the cooperative visible-light photoredox/phosphate acid catalysis (Scheme 39) [85]. However, because of two possible reaction sites adjacent to the sulfur atom, this cyanation reaction proceeded in poor regioselectivity, albeit in good to excellent yields. The phosphate acid generates the phosphate radical through one-electron photooxidation to work as a HAT catalyst, which was inspired by the natural DNA cleavage mechanism induced by photolysis.

### 2.10. C–H (Hetero)arylation

The (hetero)aryl-containing motif is one of the most common and valuable scaffolds, and C–H (hetero)arylation offers a more step-economical and greener method to synthesize (hetero)aryl derivatives [86,87].

#### 2.10.1. C(sp^3^)–H Arylation

**Scheme 40:**
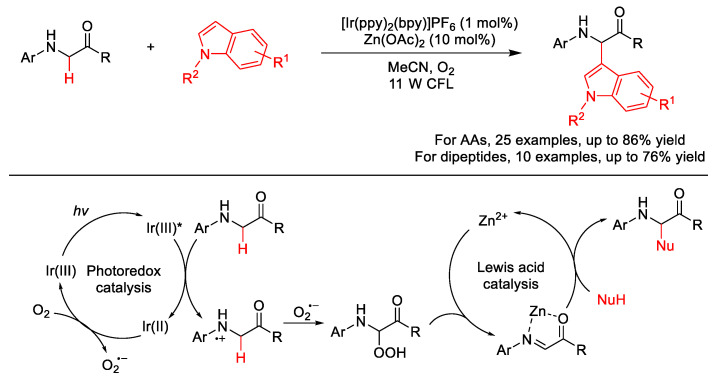
C(sp^3^)–H arylation of Gly derivatives with indoles through the cooperative catalysis of photoredox and Lewis acid.

In 2012, Rueping’s group reported the C(sp^3^)–functionalization of glycine derivatives with indoles as nucleophiles under the cooperative visible-light photoredox catalysis and Lewis acid catalysis (Scheme 40) [88]. The Lewis acid activated the hydroperoxide intermediate to form the highly electrophilic complex, which was easily attacked by the indole nucleophiles to generate the functionalized glycine derivatives. Moreover, 10 examples of dipeptides were successfully functionalized with excellent regioselectivities, and no peptide degradation was found under the mild conditions.

**Scheme 41:**
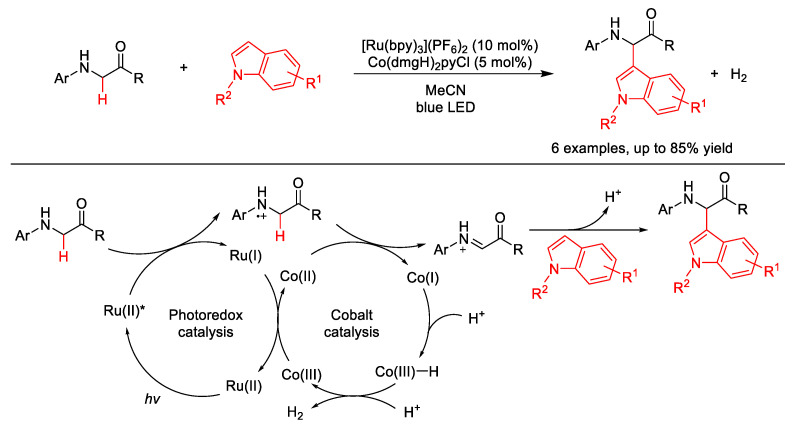
C(sp^3^)–H arylation of Gly derivatives with indoles through the cooperative catalysis of photoredox and cobalt.

Different from Rueping’s strategy of the combination of visible-light photoredox catalysis and Lewis acid catalysis, Wu’s group has merged photoredox catalysis with cobalt catalysis to realize the C(sp^3^) functionalization of glycine derivatives with indoles in excellent yields under oxidant-free and water-free conditions (Scheme 41) [39]. In this work, the cobalt catalyst was introduced to capture the electrons and protons from the photocatalyst and the substrate radical cation, respectively.

**Scheme 42:**
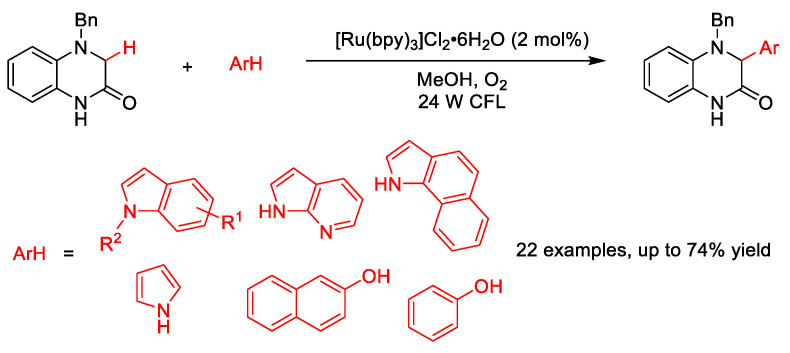
C(sp^3^)–H arylation of Gly derivatives with arenes.

Aryl nucleophiles have been further expended to 7-azaindole, 1*H*-benzo[*g*]indole, pyrrole, naphthalen-2-ol, and phenol by Hong’s group in the photoredox-mediated arylation reactions of glycine derivatives catalyzed by ruthenium complex with a household compact fluorescent light (CFL) (Scheme 42) [84]. Notably, this reaction could also occur under a catalyst-free condition with a significantly longer reaction time.

**Scheme 43:**
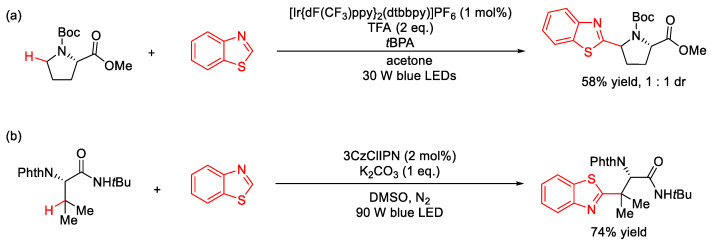
C(sp^3^)–H arylation of Pro (**a**) and Leu (**b**) derivatives with benzothiazole.

In addition, benzothiazole could also be functionalized to amino acids through the photo-mediated C(sp^3^)–H activation under mild conditions. Wang’s group [89] and Yu’s group [90] independently reported the heteroarylation of proline (Scheme 43a) and valine (Scheme 43b) in moderate yields, respectively.

**Scheme 44:**
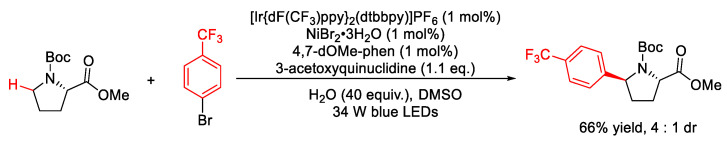
C(sp^3^)–H arylation of Pro derivative with aryl bromide.

In addition to electron-rich aryl compounds, electron-deficient aryl bromides were also employed as the arylation reagent in the photocatalytic arylation reactions. A proline derivative was arylated with 4-bromobenzotrifluoride through the combination of photoredox-mediated catalysis and nickel catalysis (Scheme 44) [91].

#### 2.10.2. C(sp^2^)–H Heteroarylation

**Scheme 45:**
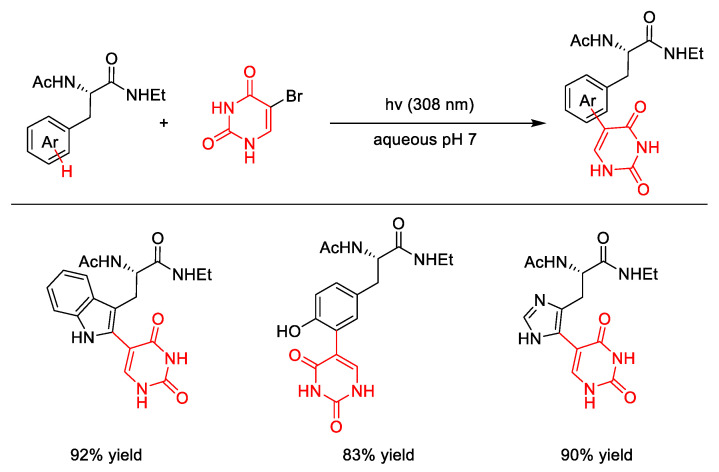
C(sp^2^)–H heteroarylation of Trp, Tyr, and His derivatives with 5-bromouracil.

In 1987, Koch’s group developed a photochemical coupling reaction between 5-bromouracil and tryptophan, tyrosine, and histidine derivatives, respectively, under the irradiation of a 400 W medium pressure Hg lamp, delivering corresponding coupling products in 83–92% yields (Scheme 45) [92]. The photocoupling reaction showed excellent regioselectivities. For instance, uracil was decorated on the 2-position of the indole of the tryptophan derivative.

**Scheme 46:**
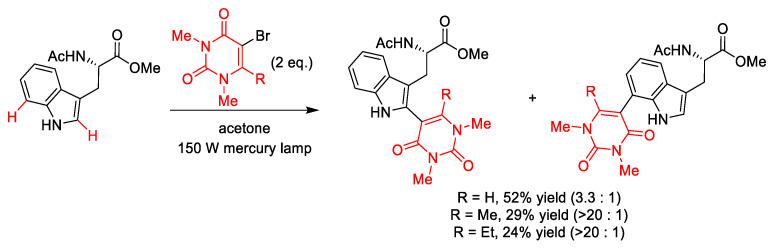
C(sp^2^)–H heteroarylation of Trp derivative with 5-bromo-1,3-dimethyluracils.

Later in 1989, Celewicz’s group has developed a photochemical coupling reaction between tryptophan and 5-bromo-1,3-dimethyluracil to produce adducts in 52% yield (Scheme 46) [93]. Interestingly, other than the 2-functionalized product, a previously unreported coupling product was characterized to be tryptophan with uracil on the 7-position of the indole. In addition, the reaction exhibited excellent regioselectivities with alkyl substituents on the 6-position of the uracil, albeit the poor reactivities.

## 3. C–N Formation

Although significant progress has been made in the realm of photo-mediated C–C formation of amino acids, the C–H nitrogenation reactions are also investigated for the practical use of the azido group [94] and the frequent occurrence of the amie or amide structure in the organic molecules [95].

### 3.1. C(sp^3^)–N Formation

**Scheme 47:**
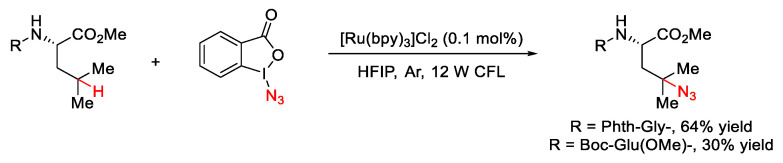
C(sp^3^)–H azidation of Leu-containing dipeptides with azidoiodane.

In 2016, Chen’s group reported an example of C(sp^3^)–azidation of leucine-containing dipeptides with azidoiodane in moderate yields under the photocatalysis by [Ru(bpy)_3_]Cl_2_ (Scheme 47) [96].

**Scheme 48:**
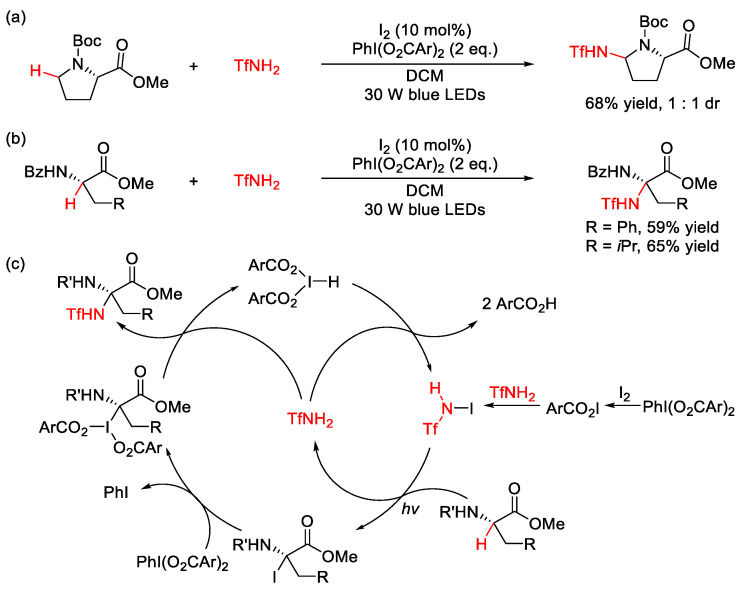
C(sp^3^)–H amination of Pro (**a**), Phe, and Leu (**b**) derivatives with triflamide. (**c**) Proposed mechanism.

A direct amination on the α-position of amino groups of amino acids under the visible-light mediation has been developed by Muñiz’s group [97]. Proline (Scheme 48a), phenylalanine, and leucine (Scheme 48b) derivatives were regioselectively aminated with triflamide (TfNH_2_), a catalytic amount of molecular iodine, and PhI(O_2_CAr)_2_ in 59–68% yields. Mechanically, ArCO_2_I was formed from PhI(O_2_CAr)_2_ and molecular iodine [98], which subsequently reacted with TfNH_2_ to generate the *N*-iodinated species. Photo-induced homolysis and the following HAT process would yield the iodo-substituted amino acids. An iodine(III) derivative was formed through an alkyl iodide oxidation phase to react with TfNH_2_ to generate the final amination product and the electrophilic (ArCO_2_)_2_IH for the next catalytic cycle (Scheme 48c).

### 3.2. C(sp^2^)–N Formation

**Scheme 49:**
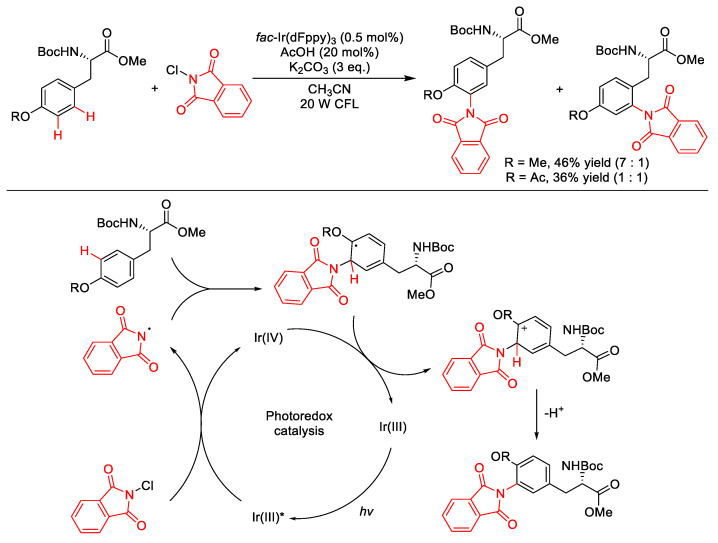
C(sp^2^)–H imidation of Tyr derivatives with *N*-chlorophthalimide.

In 2014, Lee’s group has realized the C–H imidation of tyrosine derivatives via visible-light-induced photocatalysis with *N*-chlorophthalimide (Scheme 49) [99]. Interestingly, the protecting groups on the phenols have a great influence on the regioselectivities in this reaction. The methyl group delivered products in 7:1 rr, while the acetyl group delivered products just in 1:1 rr.

**Scheme 50:**
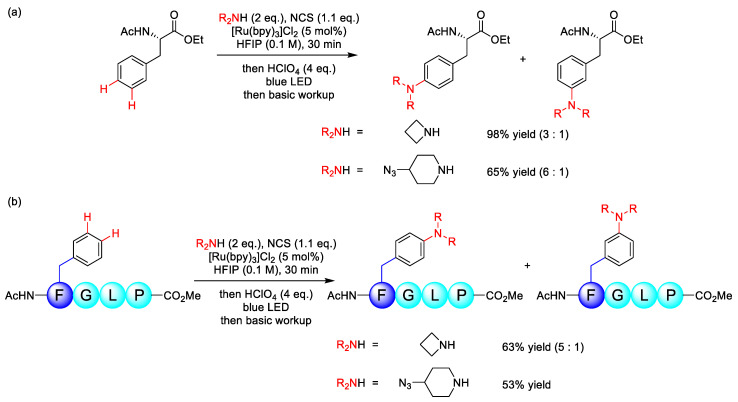
C(sp^2^)–H amination of Phe derivatives (**a**) and Phe-containing peptides (**b**) with alkyl amines.

Different from Lee’s work on C–H imidation reactions, in 2019, Leonori’s group achieved the photocatalytic C–H amination of phenylalanines using alkyl amines, which converted to *N*-chloramines in situ by the assistance of *N*-chlorosuccinimide (NCS) [100]. In this work, amination was not only applied to phenylalanines (65–98% yields) (Scheme 50a), but also tetrapeptides containing phenylalanines (53–63% yields) (Scheme 50b). Moreover, 4-azidepiperidine was compatible in the amination process and showed better regioselectivities than cyclobutylamine, providing promising chances for further functionalization.

**Scheme 51:**
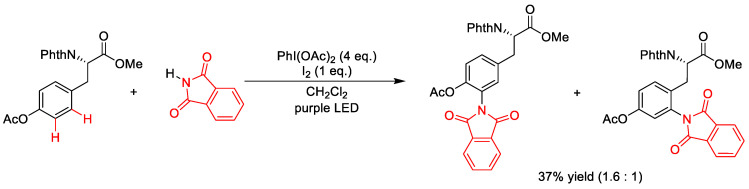
C(sp^2^)–H imidation of Tyr derivatives with phthalimide.

Recently, Nemoto and Nakajima’s group employed simple phthalimide, PhI(OAc)_2_, and molecular iodine to form *N*-iodophthalimide in situ to realize the direct metal-/photocatalyst-free C–H imidation (Scheme 51) [101]. According to the broad absorption in the UV-vis region of *N*-iodophthalimide covering 400 nm and the theoretical calculations based on density functional theory (DFT), *N*-iodophthalimide was proposed to absorb the light to generate the phthalimidyl radical for the following process.

## 4. C–S Formation

C–S bonds are privileged structures that are commonly found in natural products [102], drugs [103], and semiconductors [104]. For this reason, the construction of the C–S bond has also been widely researched due to the values of organosulfur compounds [105].

### 4.1. C(sp^3^)–S Formation

**Scheme 52:**
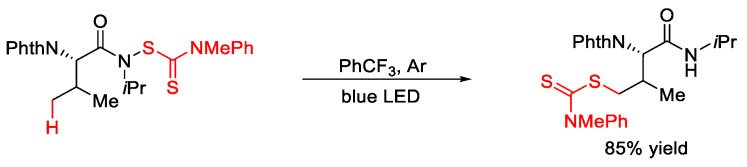
Intramolecular C(sp^3^)–H dithiocarbamylation of Val derivative.

Inspired by the intramolecular HAT reactions to functionalize the unactivated C(sp^3^)–H bonds, Alexanian’s group reported an intramolecular C(sp^3^)–H dithiocarbamylation of valine in which the dithiocarbamate worked as the transfer group (Scheme 52) [106]. Importantly, the dithiocarbamate group is an attractive pharmacophore with versatile pharmacological activities, such as antimicrobial, antifungal, antiviral, and antiparasitic activities, as well as a multifunctional precursor for allylation, deuteration, azidation, and thiolation [107,108].

### 4.2. C(sp^2^)–S Formation

**Scheme 53:**
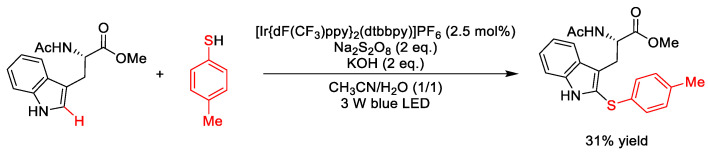
C(sp^2^)–H sulfenylation of Trp derivative with thiophenol.

A C(sp^2^)–H sulfenylation of tryptophan with thiophenol under the photocatalysis of [Ir{dF(CF_3_)ppy}_2_(dtbpy)]PF_6_ was described by Gustafson’s group in 2019 (Scheme 53) [109]. The mechanism was similar to that of the phosphonylation of tryptophan, via the attack by a nucleophilic sulfur radical, which was supported by the cyclic voltammetry and density functional theory calculations.

## 5. C–O Formation

C–H oxidation has been continuously investigated for its wide involvement in the biosynthetic pathways [110] and natural products’ total synthesis [111,112].

**Scheme 54:**
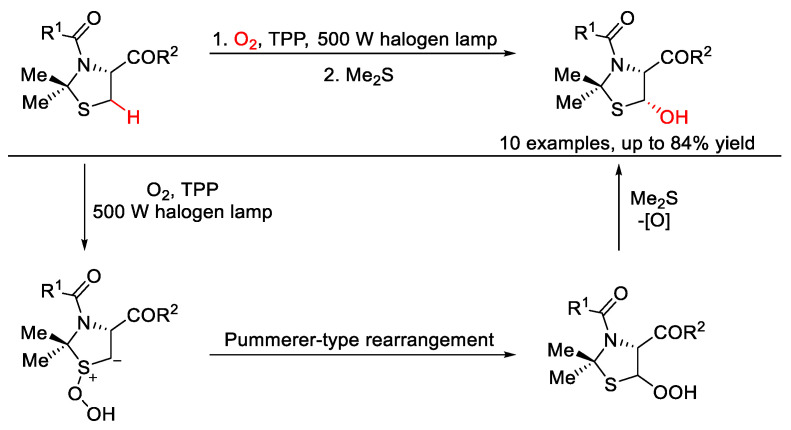
C(sp^3^)–H hydroxylation of Cys derivative.

In 2011, Long’s group has developed a photo-hydroxylation reaction of cysteine derivatives with tetraphenylporphyrin (TPP) as the photosensitizer and molecular oxygen as the oxygen source (Scheme 54) [113]. Several thiohemiacetals were obtained in moderate to good yields under the irradiation of a 500 W halogen lamp. Additionally, the thiohemiacetals could be easily further transformed to versatile bicyclic and even monocyclic β-lactams (the nucleus of penicillins), which might have promising applications for potential antibacterial activities.

**Scheme 55:**
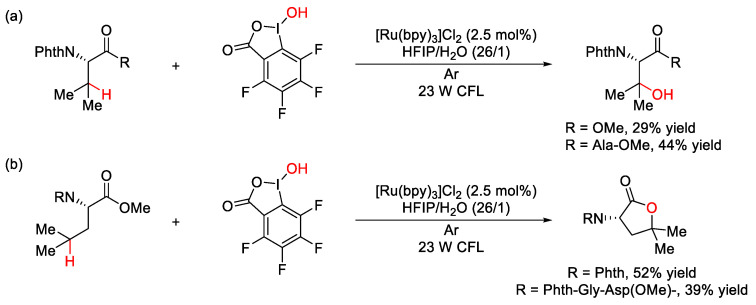
C(sp^3^)–H hydroxylation of Val (**a**) and Leu (**b**) derivatives with hydroxyl perfluorobenziodoxole (PFBl–OH).

Hydroxyl perfluorobenziodoxole (PFBl–OH) was employed as the oxygen source in the ruthenium catalyzed C(sp^3^)–H hydroxylation reaction of valine and leucine derivatives and peptides under the irradiation of 23 W CFL (Scheme 55) [114]. Interestingly, the hydroxylation of leucine derivatives would deliver lactone products in moderate yields after the spontaneous intramolecular transesterification reactions. Notably, PFBl–OH showed better results than other Bl–OH analogs for its enhanced electrophilicity of the perfluorobenziodoxole radical, which was more reactive to abstract the H atom from the substrates.

**Scheme 56:**
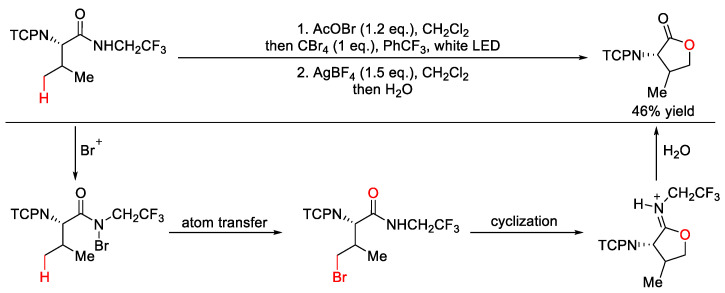
C(sp^3^)–H hydroxylation of Val derivative through tandem bromination/atom transfer/cyclization.

Tiefenbacher’s group also synthesized the lactone from the valine derivative under the irradiation of a white LED lamp (Scheme 56) [115]. The tandem reaction underwent the bromination, photo-mediated bromide transfer, intramolecular cyclization, and hydrolysis reactions, involving the brominated valine derivative as the key intermediate.

**Scheme 57:**
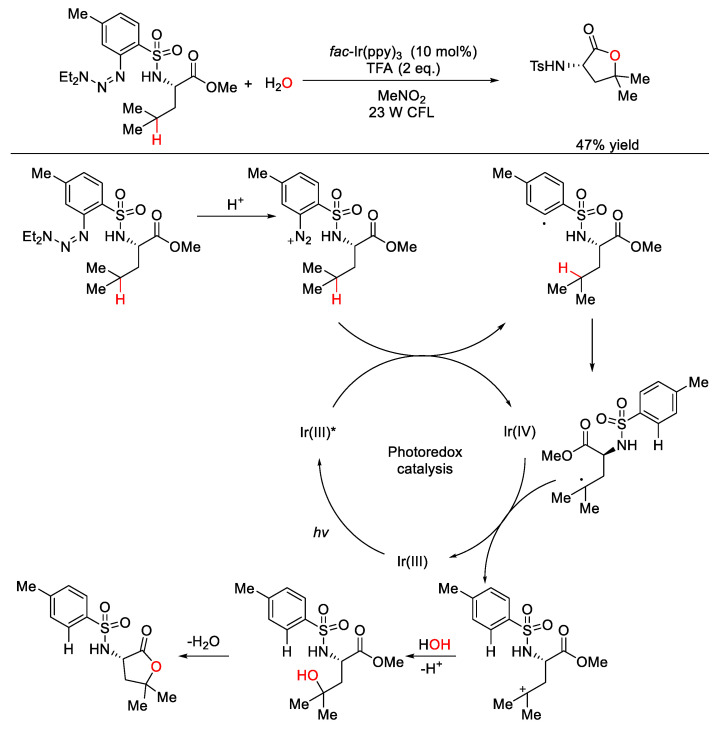
C(sp^3^)–H hydroxylation of Leu derivative with water.

Water has also been applied in the photocatalytic hydroxylation reaction of the leucine derivative by Ragains’s group (Scheme 57) [116]. The Tz^o^ group designed by Baran’s group [117] was decorated on the leucine derivative to undergo the acidification, dediazoniation, H-abstraction, oxidation, nucleophilic attack by water molecule, and the final intramolecular transesterification to give the lactone product.

## 6. C–P Formation

Phosphorus-containing organic compounds occupy an important place in pharmaceuticals [118], catalysts [119] and materials [120], resulting in the great demand of methods for the introduction of the phosphine [121]. Among the methods, the visible-light mediated direct C–H phosphorization has practical advantages for the chemical economy [122].

**Scheme 58:**
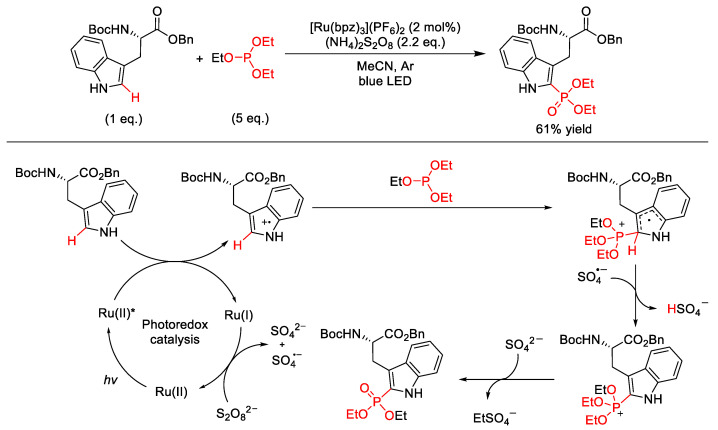
C(sp^2^)–H phosphonylation of Trp derivative with triethyl phosphite.

In 2017, König’s group developed a direct C–H phosphonylation of electron-rich heteroarenes through the photoredox catalysis of [Ru(bpz)_3_](PF_6_)_2_ and applied the method into the tryptophan in 61% yield (Scheme 58) [123]. In this reaction, triethyl phosphite attacks the radical cation of tryptophan, and then hydrogen atom abstraction by SO_4_^●−^ occurred to form the phosphonium intermediate. Finally, the rearrangement of phosphonium and SO_4_^2−^ yielded the phosphonylated tryptophan.

## 7. C–X Formation

Halogen atoms can be found in plentiful bioactive molecules [56]. Additionally, the incorporation of the halogen atoms into the lead compounds could significantly increase the drug-like properties [124,125].

### 7.1. C–H Fluorination

Selectfluor, as a commonly used fluorination reagent, has been widely investigated in the photo-induced C(sp^3^)–fluorination of amino acids. Leucine derivatives could be fluorinated at the γ-position, catalyzed by anthraquinone (AQN) [126], acetonephenone [127], and iridium complex [77], under the irradiation of the visible light (Table 1, entries 1–3). Additionally, Hamashima and Egami’s group reported a catalyst-free fluorination of leucine (Table 1, entry 4), phenylalanine (Table 1, entry 7), and valine (Table 1, entry 8) derivatives under the irradiation of an LED (365 nm) [128]. Meanwhile, phenylalanine could also be photo-fluorinated at the β-position catalyzed by 9-fluorenone (Table 1, entry 5) [129] and 1,2,4,5-tetracyanobenzene (TCB) (Table 1, entry 6) [130] in moderate yields. Moreover, Leonori and coworkers also applied the method to the isoleucine (Table 1, entry 9) and lysine (Table 1, entry 10) derivatives, albeit with unsatisfied efficiency [77].

**Scheme 59:**
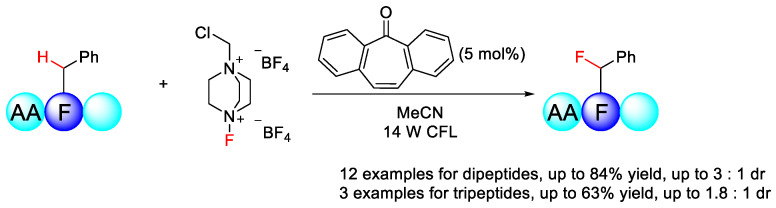
C(sp^3^)–H fluorination of Phe-containing peptides with Selectfluor.

Lectka’s group reported a phenylalanine-specific visible-light-sensitized, 5-dibenzosuberenone-catalyzed benzylic C(sp^3^)–H fluorination of phenylalanine-containing peptides with Selectfluor (Scheme 59) [131]. Interestingly, the reaction exhibited excellent intramolecular selectivity but unexpected low intermolecular selectivity, which may be ascribed to a product-determining step after the rate-determining step.

**Scheme 60:**
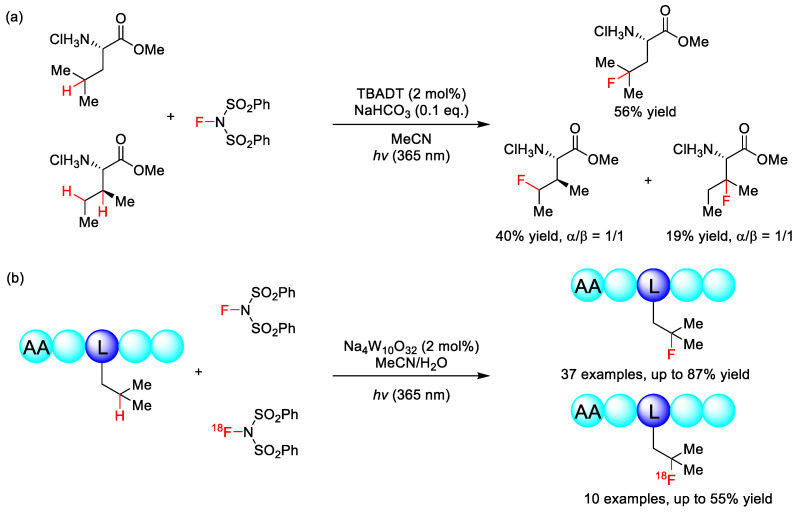
C(sp^3^)–H fluorination of Leu and Ile derivatives (**a**) and Leu-containing peptides (**b**) with *N*-fluorobenzenesulfonimide (NSFI).

In 2014, Britton’s group developed a photocatalytic fluorination reaction of leucine and isoleucine derivatives with *N*-fluorobenzenesulfonimide (NSFI) as the mild fluorine atom transfer reagent, which was catalyzed by the easily prepared tetrabutylammonium decatungstate (TBADT) (Scheme 60a) [132]. Based on this work and the study on the uniquely susceptible property to C–H abstraction [133,134,135], Britton, Schaffer, and coworkers [136] further investigated the photocatalytic fluorination reaction of Leu-containing peptides, and 37 examples of reaction gave high yields up to 87%. Importantly, the isotope ^18^F could also be successfully labeled in 10 peptides, which had great positive impact on the discovery of radiolabeled peptides as imaging agents and peptide-based drugs (Scheme 60b).

### 7.2. C–H Chlorination and Bromination

**Scheme 61:**
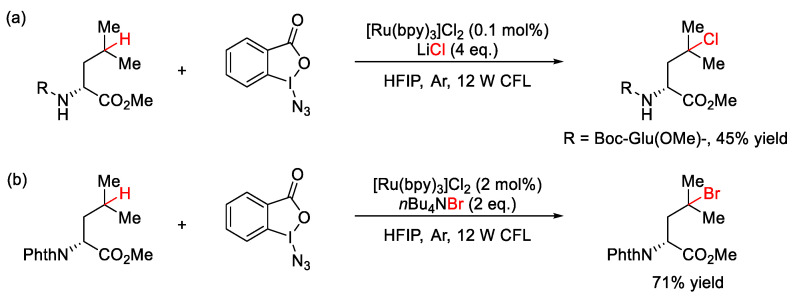
C(sp^3^)–H chlorination (**a**) and bromination (**b**) of Leu derivatives with Zhdankin reagent.

In 2016, Chen’s group developed the visible-light-promoted halogenation reactions with the Zhdankin azidoiodane reagent and ruthenium catalyst [96]. When LiCl was used as the additive, the dipeptide of leucine could be chlorinated at the γ-position in moderate yield (Scheme 61a). While when the additive was altered to *n*Bu_4_NBr, the bromination product of leucine derivative could be achieved in 71% yield (Scheme 61b).

**Scheme 62:**
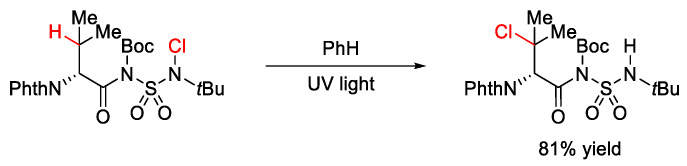
Intramolecular C(sp^3^)–H chlorination of Val derivative.

In 2020, Roizen’s group installed a *N*-chlorosulfamide group to the valine derivative (Scheme 62) [137]. The substrate could proceed with chlorine-atom abstraction, 1,6-HAT, and the final chlorination process to finish the formal 1,6-chlorine-atom transfer reaction.

## 8. Conclusions

This review has described the direct synthesis of NPAAs from PAAs through the photo-mediated C–H functionalization reactions. Furthermore, on account of the mild conditions, high competence, broad functional group tolerance, and great energy-saving performance, photo-mediated C–H functionalization reactions have been considered as powerful methods to modify peptides with plentiful and valuable functional groups. Hopefully, the elegant pioneering works reviewed here would serve as a solid base and provide guidance to the development of more photoreactions to deliver more versatile bioactive functionalized amino acids and peptides under mild conditions.

## Figures and Tables

**Table 1:** C(sp^3^)–H fluorination of amino acids with Selectfluor.

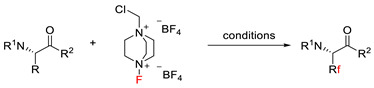					
**Entry**	**Substrates**	**Products**	**Conditions**	**Yields**	**Ref.**
1	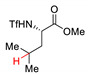	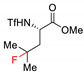	AQN (2 mol%) O_2_ free MeCN 11 W CFL	34%	[125]
2			acetophenone (20 mol%) MeCN 19 W CFL	85%	[126]
3			Ir[dF(CF_3_)ppy]_2_(dtbbpy)PF_6_ (5 mol%) Cs_2_CO_3_ (1 eq.) MeCN/H_2_O blue LED	5 examples up to 56%	[77]
4			MeCN LED (365 nm)	45%	[127]
5	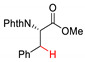	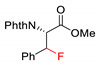	9-fluorenone (5 mol%) MeCN 11 W CFL	52% 1:1 dr	[128]
6			TCB (10 mol%) MeCN *hv* (302 nm)	62% 1:1 dr	[129]
7			MeCN LED (365 nm)	43% 1:1 dr	[127]
8	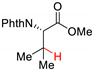	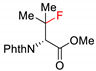	MeCN *hv* (365 nm)	70%	[127]
9	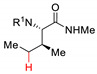	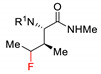	Ir[dF(CF_3_)ppy]_2_(dtbbpy)PF_6_ (5 mol%) Cs_2_CO_3_ (1 eq.) MeCN/H_2_O blue LED	39% 1.5:1 dr	[77]
10	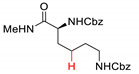	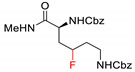	Ir[dF(CF_3_)ppy]_2_(dtbbpy)PF_6_ (5 mol%) Cs_2_CO_3_ (1 eq.) MeCN/H_2_O blue LED	25%	[77]
